# Circular SNX25 encoded radioresistance augmenter facilitates DNA damage repair in hepatocellular carcinoma by targeting BAG6-GET4 interaction

**DOI:** 10.1038/s41419-025-08026-9

**Published:** 2025-10-21

**Authors:** Shuping Li, Ying Gao, Yuhao Tang, Aoran Dong, Shuifang Hu, Ruizhi Wang, Xiaoyong Gong, Zhenwei Peng

**Affiliations:** 1 Department of Radiation Oncology, The First Affiliated Hospital of Sun Yat-sen University, Guangzhou, China; 2 Cancer Center, The First Affiliated Hospital of Sun Yat-sen University, Guangzhou, China; 3 Laboratory of General Surgery, The First Affiliated Hospital, Sun Yat-sen University, Guangzhou, China; 4 Department of liver surgery, The Sun Yat-sen University Cancer Center, Guangzhou, China; 5 Digestive Diseases Center, The Seventh Affiliated Hospital of Sun Yat-sen University, Shenzhen, China; 6 Department of Laboratory Medicine, The First Affiliated Hospital of Sun Yat-sen University, Guangzhou, China; 7 Department of General Surgery, Ruijin Hospital, Shanghai Jiao Tong University School of Medicine, Shanghai, China; 8 Department of General Surgery, Ruijin-hainan Hospital, Shanghai Jiao Tong University School of Medicine, Qionghai, China; 9 Institute of Precision Medicine, The First Affiliated Hospital of Sun Yat-sen University, Guangzhou, China; 10 Clinical Trials Unit, The First Affiliated Hospital of Sun Yat-sen University, Guangzhou, China

**Keywords:** Radiotherapy, Prognostic markers, DNA damage and repair, Non-coding RNAs, Predictive markers

## Abstract

Radiotherapy (RT) is a crucial treatment for hepatocellular carcinoma (HCC); however, resistance to radiation remains a major challenge to its effectiveness. Circular RNAs (circRNAs), traditionally considered stable non-coding RNAs, have recently gained attention for their potential to encode proteins or peptides that play crucial roles in HCC radioresistance. In this study, circSNX25 (has_circ_0004874) was identified as a novel circRNA with coding potential related to radiation resistance in HCC through circRNA sequencing, Northern blotting, and mass spectrometry (MS). We demonstrated that SNX25-215, a novel protein encoded by circSNX25, rather than the parental circSNX25 itself, promotes resistance to radiotherapy in HCC cells both in vitro and in vivo. Co-immunoprecipitation (co-IP) and molecular docking revealed that amino acids H207 and E214 of SNX25-215 are critical for binding to Golgi to ER traffic protein 4 homolog (GET4). This interaction inhibits the binding of BCL2-associated athanogene 6 (BAG6) to GET4, thereby exposing the nuclear localization sequence (NLS) of BAG6 and facilitating its nuclear translocation. This, in turn, enhances DNA damage repair, ultimately increasing resistance to ionizing radiation (IR) in HCC cells. Importantly, elevated levels of SNX25-215 lead to nuclear localization of BAG6, endowing HCC cells with radioresistant activity, which is further supported by clinical evidence. Our findings highlight the potential of circSNX25 as a prognostic biomarker and a therapeutic target for overcoming radioresistance in HCC. This study provides deeper insights into the roles that circRNA-encoded proteins play in HCC radioresistance.

## Introduction

Hepatocellular carcinoma (HCC), accounting for 75–85% of primary liver cancers, is the sixth most prevalent cancer globally and the third leading cause of cancer-related mortality [1]. Radiotherapy (RT) is a key modality for the local treatment of HCC, effectively enhancing local control and improving patient survival [2]. However, its clinical efficacy is limited by radiation resistance in HCC cells [3, 4]. A deeper understanding of the mechanisms underlying radioresistance is crucial for identifying novel biomarkers and therapeutic targets to improve radiotherapy outcomes in HCC patients.

Circular RNAs (circRNAs) are covalently closed RNA molecules known for their high stability [5], and their aberrant expression in various cancers highlights their potential as diagnostic tools, therapeutic targets, or biomarkers [6]. Although circRNAs were traditionally considered non-coding RNAs, recent studies have revealed that a subset of circRNAs contain open reading frames (ORFs) and can recruit ribosomes, suggesting their capacity to encode proteins [7]. Notably, circRNA-encoded proteins, characterized by their prolonged expression, have been implicated in critical cancer-related processes, including tumor cell proliferation, migration, invasion, and treatment response [8]. For instance, circASK1 and circATG4B encode proteins that confer resistance to gefitinib in lung adenocarcinoma and oxaliplatin in colorectal cancer, respectively [9, 10], while circAKT3 encodes a protein associated with radiation resistance in glioblastoma [11]. These findings suggest that circRNA-encoded proteins may play an important role in HCC radiotherapy resistance, though research in this area remains limited, emphasizing the need for further investigation.

Ionizing radiation (IR) kills tumor cells primarily by inducing DNA damage, particularly lethal double-strand breaks (DSBs), which simultaneously activate a series of cellular DNA damage responses (DDRs). However, tumor cells can develop resistance to radiotherapy by enhancing these protective DDRs, including DNA damage sensing, early signal transduction, cell cycle arrest, and DNA repair [12]. Among these, the p53/p21 pathway plays a critical role in regulating cell cycle checkpoints, particularly G1/S arrest, to delay cell cycle progression and provide additional time for DNA repair [13]. Notably, BCL2-associated athanogene 6 (BAG6), a multifunctional protein predominantly localized in the cytoplasm [14], becomes essential for DDRs when translocated to the nucleus [15]. In the nucleus, BAG6 interacts with p300 to facilitate p53 acetylation, a modification required for p53-mediated DNA damage responses [16]. DSBs generated by IR are primarily repaired through either nonhomologous end-joining (NHEJ) or homologous recombination (HR) pathways [13]. In NHEJ repair, nuclear BAG6 interacts with disruptor of telomeric silencing 1-like (DOT1L) to facilitate H3K79 di-methylation, which is essential for the formation of p53-binding protein 1 (53BP1) foci, a critical step in the NHEJ repair pathway [17]. In HR repair, nuclear BAG6 forms complexes with breast cancer type 1 susceptibility protein (BRCA1) and localizes to DNA damage sites, promoting the formation of BRCA1 foci to enhance HR repair efficiency [18]. Moreover, BAG6 stabilizes SET domain containing protein 1 A (SET1A) activity, increasing H3K4 di-methylation at the BRCA1 promoter and thereby enhancing its expression [19]. These findings highlight the pivotal role of nuclear BAG6 in coordinating multiple DDRs mechanisms, ultimately contributing to radiotherapy resistance.

In this study, we identified circSNX25, a protein-coding circRNA upregulated in radioresistant HCC patients and closely associated with poor prognosis. Mechanistically, circSNX25 encodes a previously unidentified protein consisting of 215 amino acids, designated as SNX25-215. Both in vivo and in vitro experiments demonstrated that SNX25-215, but not circSNX25 itself, enhances radioresistance in HCC cells. The Golgi to ER traffic protein 4 homolog (GET4) was identified as a key interaction partner of SNX25-215. Previous studies have shown that GET4 (residues 195–271) masks the nuclear localization sequence (NLS) of BAG6, thereby preventing its translocation into the nucleus [20, 21]. We further discovered that SNX25-215 competes with BAG6 for binding to GET4 (residues 195–271), exposing BAG6’s NLS and facilitating its nuclear translocation, which enhances DDRs upon IR exposure. Clinically, high levels of circSNX25 and its encoded protein SNX25-215 may both serve as prognostic factors for poor overall survival (OS) and progression-free survival (PFS) in IR-treated HCC patients. Collectively, our findings suggest that circSNX25 contributes to radioresistance in HCC by encoding the novel protein SNX25-215, highlighting its potential as a prognostic biomarker and a therapeutic target to improve radiotherapy outcomes of HCC patients.

## Materials and methods

### Cell culture

HCC cell lines MHCC-97H, HCCLM3, HCCLM9, Huh7, PLC/PRF/5 and SNU-449 were purchased from the Cell Bank of the Shanghai Institutes of Biological Sciences (Shanghai, China). The human embryonic kidney 293T (HEK293T) cell line was purchased from the American Type Culture Collection (ATCC). All cell lines were cultured in Dulbecco’s modified Eagle’s medium (DMEM), supplemented with 10% fetal bovine serum (FBS) and 1% penicillin-streptomycin. Cell lines were routinely tested for mycoplasma contamination and authenticated using short tandem repeat (STR) profiling.

### CircRNA sequencing

CircRNA sequencing of HCC tissues and their matched normal counterparts was conducted by Ribo Biotechnology (Guangzhou, China). Differentially expressed circRNAs (DEcircRNAs) were identified using a fold change threshold greater than 2 or less than 0.5 and a p-value less than 0.05.

### Human HCC samples

Histopathological and clinical diagnoses were confirmed for all HCC specimens utilized in this study. Informed consent was obtained from all patients prior to enrollment, and the study protocol was approved by the Institutional Research Ethics Committee of Sun Yat-sen University First Affiliated Hospital (SYSUFAH) (Approval No. [2022]438). All procedures involving human participants adhered to relevant ethical regulations.

Forty-eight patients with a histologically confirmed diagnosis of HCC at SYSUFAH prior to receiving radical radiotherapy (50 Gy in 25 fractions) were enrolled in this study. All patients received contrast-enhanced CT or MRI scans both before and after radiotherapy (RT). Tumors response was assessed according to the Modified Response Evaluation Criteria in Solid Tumor [22]. Briefly, tumors were evaluated within 6 months after the completion of RT. If a reduction in tumor volume, loss of the “fast-in and fast-out” phenomenon, or local tumor inactivation (no obvious enhancement in the arterial phase) was observed, the patients were classified as “radiosensitive”. Conversely, if tumor volume increased or local recurrences or new lesions appeared in the liver, patients were classified as “radioresistant”.

Additionally, ten specimens were obtained from HCC patients who received RT followed by either palliative surgery or needle biopsy upon tumor recurrence.

Overall survival (OS) was defined as the time between initial HCC diagnosis and death or the date of the last follow-up. Progression-free survival (PFS) was defined as the time from the completion of radiotherapy to disease progression or death. Following radiotherapy at SYSUFAH, patients were monitored one month after treatment completion, with subsequent follow-up assessments every three months until disease progression or data censoring.

### Total RNA and protein extraction from Formalin-Fixed, Paraffin-Embedded (FFPE) tissue sections

Total RNA of FFPE tissues from forty-eight human HCC samples was extracted using the DynaPure XP® AllPrep DNA/RNA FFPE Kit (bphealth, Hangzhou, China) according to the manufacturer’s protocols. Total protein of FFEP tissues from ten human HCC samples was extracted using the FFPE Total Protein Extraction Kit (Sangon Biotech, Shanghai, China) following the manufacturer’s instructions.

### In vivo studies

All animal care and experiment protocols were approved by the Clinical Research and Animal Trials of the First Affiliated Hospital of Sun Yat-sen University (Approval No. [2023]124). The mice were treated in accordance with the National Institutes of Health (NIH) guidelines for the care and use of laboratory animals.

To establish a subcutaneous xenograft mouse model, 1 × 10^7^ cells were implanted subcutaneously into the flanks of NOD CRISPR *Prkdc Il2r Gamma* (NCG) mice (6–8-week-old male). Tumor volumes were measured using digital vernier calipers and calculated using the formula: (length × width^2^)/2. Once tumors reached a volume of 150–200 mm^3^, mice were randomly assigned into the indicated groups (n = 5 per group) and ionizing radiation (IR) was administered at a dose of 4 Gy per day for 3 consecutive days. To minimize radiation exposure to healthy tissues, only the tumor area was irradiated, while the rest of the body was shielded by a lead plate. Two weeks after IR, the animals were euthanized by cervical dislocation, and the tumors were excised and weighed.

### RNA isolation, cDNA synthesis and quantitative PCR

Total RNA was extracted from cells using the TRIzol reagent (Thermo Fisher Scientific, MA, USA) according to the manufacturer’s instructions. Complementary DNA (cDNA) was synthesized from the extracted RNA using the GoScript Reverse Transcription System (Promega, WI, USA). Gene expression was quantified by quantitative PCR (qPCR) using the RealUniversal Color PreMix (TIANGEN, Beijing, China) following the manufacturer’s protocols. Glyceraldehyde-3-phosphate dehydrogenase (GAPDH), beta-actin, U3 and U6 were used as endogenous reference for data normalization. The specific primer sequences used are listed in Supplementary Table S1.

### Northern blotting

Briefly, 20 µg RNA was mixed with 5 μL of loading buffer, incubated at 75 °C for 10 min, and then rapidly cooled on ice for 2 min. Samples were then subjected to electrophoresis at 100 V for approximately 1 h, followed by transfering to a membrane at 390 mA for 40–60 min. The RNA was crosslinked on the membrane using a UV crosslinker for 2–10 min. Hybridization was performed with a digoxigenin (DIG)-labeled probe specific to the circSNX25 back-splicing junction at 68 °C for 18 h (Sequences listed in Supplementary Table S2). The membrane was then incubated with an anti-DIG solution for 30 min, followed by detection using a chemiluminescent substrate and visualization with a gel imaging system.

### RNase R treatment

Total RNA (2.5 µg) was either left untreated (control) or subjected to RNase R digestion. For the control, 2.5 µg of total RNA was mixed with 15.5 µL of RNase-free water and 2 µL of 10× RNase R Reaction Buffer (Geneseed Biotechnology, Guangzhou, China). For the RNase R treatment, 0.125 µL of RNase R (20 U/µL) was added to the same mixture. Both samples were then incubated at 37 °C for 30 min before proceeding to reverse transcription. SNX25 mRNA was used as an internal control.

### Subcellular fractionation

RNA and protein were extracted from nuclear and cytoplasmic fractions using the Minute Cytoplasmic and Nuclear Extraction Kits (Invent Biotechnology, MN, USA), following the manufacturer’s protocols.

### Co-immunoprecipitation (Co-IP) and mass spectrometry (MS) analysis

For co-immunoprecipitation (Co-IP), cells were transfected with the indicated plasmid. Twenty-four hours post-transfection, the proteasome inhibitor MG132 was added to a final concentration of 10 µM, and the cells were incubated overnight. Cells were collected by centrifugation and lysed in cell lysis buffer (Cell Signaling Technology, MA, USA) supplemented with protease and phosphatase inhibitors. The lysates were incubated overnight with specific antibodies at 4 °C. Subsequently, protein A/G beads (Smart-Lifesciences, Changzhou, China) were added to the mixtures and incubated for 30 min at room temperature. After thorough washing, immunoprecipitated proteins were eluted from the beads and resolved by sodium dodecyl sulfate-polyacrylamide gel electrophoresis (SDS-PAGE).

HCCLM9 cells transfected with FLAG-tagged circSNX25, FLAG-tagged SNX25-215 or vector control were subjected to IP using FLAG magnetic beads (Sigma-Aldrich, MO, USA) overnight at 4 °C. The beads were then washed with wash buffer (25 mM Tris-HCl pH 7.4, 150 mM NaCl, 1 mM EDTA, 1% NP-40, 5% glycerol), followed by elution with 1 M glycine (pH 3.0). A portion of the bead-bound protein complex was then directly subjected to mass spectrometry (MS) analysis, which was performed by Huada Gene (Shenzhen Huada Gene Research Institute, Shenzhen, China).

### Polysome profiling

Cells were lysed in 425 µL of lysis buffer containing 5 mM Tris-HCl (pH 7.5), 1.5 mM KCl, 2.5 mM MgCl_2_, and 1× protease and phosphatase inhibitors. Subsequently, 5 µL of 10 mg/mL cycloheximide, 1 µL of 1 M dithiothreitol (DTT), and 100 units of RNasin (Promega, WI, USA) were added. The mixture was vortexed for 5 s, then 25 µL of 10% Triton X-100 and 25 µL of 10% sodium deoxycholate were added, followed by another 5-s vortexing. The lysate was then centrifuged for 2 h at 4 °C in a continuous density gradient centrifuge using a sucrose gradient (5%–50%), prepared in a buffer containing 20 mM HEPES (pH 7.6), 100 mM KCl, 5 mM MgCl_2_, 10 µg/mL cycloheximide, 1× protease inhibitor cocktail, and 10 units/mL RNase inhibitor. The centrifuged product was fractionated into 8 equal parts using a fully automated density gradient preparation and separation instrument (Brandel, MD, USA), which also generated continuous absorbance profiles. RNA was extracted from the isolated fractions using TRIzol reagent, followed by reverse transcription and qPCR.

### Luciferase reporter assay

Luciferase activity was measured using the Dual-Luciferase Assay Kit (Promega, WI, USA). Briefly, cells were seeded at a density to reach 70–80% confluency after 24 h. The cells were then transfected with the indicated plasmids, including the P-Luc2-IRES-Report vector (Geneseed Biotechnology, Guangzhou, China) and the specified expression constructs. After transfection, the cells were maintained under standard growth conditions for additional 24 h. The cells were then harvested, washed with phosphate-buffered saline (PBS), and lysed using the lysis buffer provided in the luciferase assay kit. Following a brief incubation to ensure complete lysis, the lysates were carefully transferred to a 96-well plate. Luciferase activity was measured according to the manufacturer’s protocol using an Infinite F500 plate reader (TECAN, Switzerland).

### Immunofluorescence

HCC cells were seeded onto coverslips and allowed to adhere. Subsequently, the cells were fixed with 4% paraformaldehyde (Beyotime, Shanghai, China) for 15 min. Following permeabilization with 0.5% Triton X-100 for 15 min, cells were incubated with primary antibodies overnight at 4 °C. Cells were then incubated with fluorophore-conjugated secondary antibodies and stained with 4′,6-diamidino-2-phenylindole (DAPI) (Beyotime, Shanghai, China) to visualize nuclei. Immunofluorescence was visualized using a confocal laser scanning microscope (OLYMPUS IX83-FV3000). Antibody details are listed in Supplementary Table S2.

### Immunohistochemistry and peptide blocking experiment

Paraffin-embedded tissue sections were deparaffinized and rehydrated using standard protocols. Antigen retrieval was performed to unmask epitopes, followed by blocking of endogenous peroxidase activity. The sections were then incubated overnight at 4 °C with the primary antibodies. Subsequently, the sections were incubated with a horseradish peroxidase (HRP)-conjugated secondary antibody and visualized using 3,3′-diaminobenzidine (DAB) chromogen. Finally, the sections were counterstained with hematoxylin to visualize nuclei.

The protocol for pre-absorption of the primary antibody with its corresponding antigen involved incubating the antibody at room temperature for 4 h with 200 µg of peptide per mL of antibody at the working concentration. The working concentration of the antibody used is listed in Supplementary Table S2.

### Vector construction and cell transfection

Wild-type (WT) or mutant circSNX25 was cloned into the pLO-ciR vector by Geneseed Biotechnology (Guangzhou, China). GFP-fused SNX25-215 plasmid was constructed by Genecreate (Wuhan, China). The coding sequences for SNX25-215-FLAG or mutant SNX25-215-FLAG were inserted into the pCDH-CMV-MCS-EF1-copGFP vector (Tsingke, Beijing, China). The coding sequences for BAG6-HA, GET4-FLAG, and full-length or truncated GET4-HA were inserted into the pCDH-CMV-MCS-EF1-RFP-T2A-Puro vector (Tsingke, Beijing, China).

Stable cell lines were generated through retroviral infection followed by antibiotic selection or sorting of cells with high GFP expression by flow cytometry. Scrambled siRNA (si-NC) and siRNAs targeting specific genes (Sequences listed in Supplementary Table S2) were purchased from Ribobio (Guangzhou, China). Cells were transfected with 50 nM siRNA using Lipofectamine RNAiMAX (Thermo Fisher Scientific, MA, USA) according to the manufacturer’s protocols.

### Colony formation assay

Cells were plated in triplicate in 6-well or 12-well plates and incubated overnight. After irradiation with a linear accelerator (RS2000, RadSource, Suwanee, USA) at doses ranging from 0 to 6 Gy, cells were returned to the incubator and cultured for 10–20 days, with the medium being replaced every 3 days. Colonies were fixed with 4% formaldehyde and stained with 0.5% crystal violet. Colony counts were analyzed using ImageJ software. The plating efficiency (PE) was calculated as the number of colonies formed divided by the number of seeded cells, multiplied by 100%. The survival fraction was determined by dividing the number of colonies at a given irradiation dose by the number of colonies at 0 Gy, corrected for PE.

### Western blotting

Cells were lysed using RIPA lysis buffer (Thermo Fisher, MA, USA) supplemented with protease and phosphatase inhibitor cocktails (Beyotime, Shanghai, China). Protein concentrations were determined using a BCA kit (Thermo Fisher Scientific, MA, USA). The cell lysates were then subjected to SDS-PAGE and transferred onto a polyvinylidene fluoride (PVDF) membrane (Millipore, MA, USA). The membrane was probed with primary antibodies, followed by HRP-conjugated secondary antibodies. A high-sensitivity ECL western blotting substrate (Tanon) was used to visualize the signal. Details of the antibodies used are provided in Supplementary Table S2.

### 3D modeling and molecular docking

De novo modeling of human SNX25-215 and GET4 was performed using AlphaFold2 by Wecomput Technology (Beijing, China). Molecular docking simulations were conducted with ClusPro2 to evaluate the most likely protein-protein interactions based on intermolecular contacts. The resulting complex models and their interface residues were further analyzed using MOE (Chemical Computing Group). Molecular graphics were generated using PyMOL (Schrödinger).

### Statistical analysis

Data were obtained from three independent experiments and presented as mean ± standard deviation (SD). Statistical significance between two groups was determined using Student’s t-tests. One-way or two-way analysis of variance (*ANOVA*) was employed for multigroup comparisons. Correlations were assessed using Spearman or Pearson correlation analysis. All statistical analyses were performed using GraphPad Prism (Version 8.0). A p-value of less than 0.05 was considered statistically significant. Transcirc (https://www.biosino.org/transcirc/) [23], circBank (http://www.circbank.cn/) [24], and circRNADb (http://reprod.njmu.edu.cn/cgi-bin/circrnadb/circRNADb.php) [25] were utilized to analyze translation potential of circSNX25.

## Results

### Identification and characterization of circSNX25 in radiation-resistant HCC

**Fig. 1:**
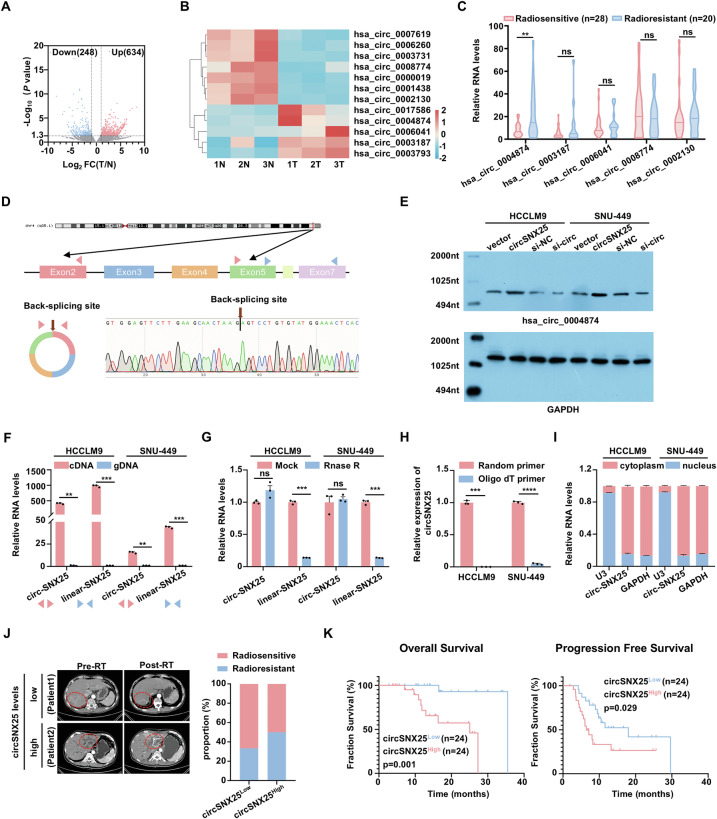
Identification and characterization of circSNX25 in radiation-resistant HCC. **A** Volcano plot showing differentially expressed circular RNAs (DEcircRNAs) between there paired hepatocellular carcinoma (HCC) and adjacent normal tissues, with fold change (FC) indicated. **B** Heatmap showing DEcircRNAs with high expression levels and consistent trends across all tissue samples. **C** Quantitative polymerase chain reaction (qPCR) analysis of 5 DEcircRNAs in 28 radiosensitive and 20 radioresistant HCC tissue samples. **D** Schematic of circSNX25 formation, with the back-splicing junction confirmed by Sanger sequencing (brown arrow). **E** Northern blotting analysis using a junction-specific circular probe to detect circSNX25 in HCCLM9 and SNU-449 cells, with and without circSNX25 overexpression or siRNA knockdown. **F** qPCR analysis of circSNX25 and linear SNX25 in complementary DNA (cDNA) and genomic DNA (gDNA) using divergent and convergent primers. **G** Stability comparison of circSNX25 versus linear SNX25 after RNase R treatment, as assessed by qPCR. **H** qPCR evaluation of circSNX25 expression levels using cDNA synthesized with either oligo(dT) primers or random primers. **I** Subcellular localization of circSNX25 in HCC cells, with glyceraldehyde-3-phosphate dehydrogenase (GAPDH) and U3 as cytoplasmic and nuclear markers, respectively. **J** Representative computed tomography (CT) scans of HCC patients in the SYSUFAH cohort before and after radiotherapy (RT), stratified by high or low circSNX25 expression (left). Stacked bar chart illustrating the distribution of radiotherapy-sensitive and radiotherapy-resistant cases in low (n = 24) and high (n = 24) circSNX25 expression groups (right). **K** Kaplan–Meier analysis of overall survival (OS) and progression-free survival (PFS) in 48 HCC patients who received surgical resection or liver biopsy before radiotherapy, stratified by high (n = 24) versus low (n = 24) circSNX25 expression in the SYSUFAH cohort. Quantitative data represent mean ± SD (n = 3 independent experiments) and statistical significance was assessed by two-tailed unpaired Student’s *t* test (**C**, **F**–**H**) or log-rank test (**K**). (****p < 0.0001, ***p < 0.001, **p < 0.01, ns: not significant).

To identify circRNAs associated with radiation resistance in HCC, we performed circRNA sequencing on three pairs of HCC and adjacent normal liver tissues. A total of 882 differentially expressed circRNAs (DEcircRNAs) were identified, with 634 upregulated and 248 downregulated, as annotated in the circBase database (Fig. 1A). Among these, we selected 12 DEcircRNAs (5 upregulated, 7 downregulated) that exhibited high expression levels and consistent trends across all three tissue pairs (Fig. 1B). Of these, 5 circRNAs were successfully amplified using specific divergent primers targeting the head-to-tail junction in HCCLM9 and SNU-449 cell lines (Fig. S1A). To further investigate their association with radiation resistance, we analyzed the expression of these 5 circRNAs in 28 radiosensitive and 20 radioresistant HCC tissues. Notably, hsa_circ_0004874 exhibited significantly higher expression in tumors from radioresistant patients compared to those from radiosensitive patients, while the other 4 circRNAs showed no significant differences (Fig. 1C). Additionally, hsa_circ_0004874 was more frequently upregulated in HCC tissues than in adjacent non-cancerous tissues (Fig. S1B, C). Based on these findings, we selected hsa_circ_0004874 for further study, as its expression was strongly correlated with radiotherapy resistance in HCC.

According to circBase (http://www.circbase.org/), hsa_circ_0004874 is derived from exons two to five of the Sorting Nexin-25 (SNX25) gene located on chromosome 4, comprises 662 nucleotides (nt), and is designated as circSNX25. The head-to-tail splicing of circSNX25 was validated by Sanger sequencing (Fig. 1D). We then examined circSNX25 expression in various HCC cell lines and selected HCCLM9 and SNU-449 for further investigations (Fig. S1D). To further confirm the endogenous presence of circSNX25, Northern blotting was performed using a probe against the back-spliced junction. A 662-nt band was detected in both HCCLM9 and SNU-449 cells, with band intensity increasing upon circSNX25 overexpression and decreasing upon its knockdown (Fig. 1E).

To ensure that circSNX25 arises from genuine head-to-tail splicing rather than trans-splicing or genome rearrangement, we employed convergent and divergent primers to amplify linear SNX25 and circSNX25, respectively. Subsequently, qPCR analysis confirmed the presence of both transcripts in cDNA but not in genomic DNA (gDNA) (Fig. 1F). Moreover, circSNX25 exhibited greater resistance to RNase R digestion compared to linear SNX25, confirming its circular nature (Fig. 1G). In addition, circSNX25 was nearly undetectable when cDNA was synthesized using oligo(dT) primers, indicating its lack of a poly(A) tail (Fig. 1H). Given that the function of circRNAs is often linked to their subcellular localization [26], we investigated the distribution of circSNX25 and found it predominantly localized in the cytoplasm of HCC cells (Fig. 1I).

To assess the clinical significance of circSNX25, we stratified 48 HCC patients into high- and low-expression groups based on its median expression level. A higher proportion of patients with elevated circSNX25 expression exhibited radioresistance (Fig. 1J). Furthermore, higher circSNX25 expression was associated with significantly worse overall survival (OS) (p = 0.001) and progression-free survival (PFS) (p = 0.029) in radiotherapy-treated patients, suggesting its potential as a prognostic biomarker (Fig. 1K). Together, these results demonstrate that circSNX25 is a stable, cytoplasm-localized circRNA closely correlated with the radiation response in HCC patients.

### CircSNX25 encodes a novel protein SNX25-215

**Fig. 2:**
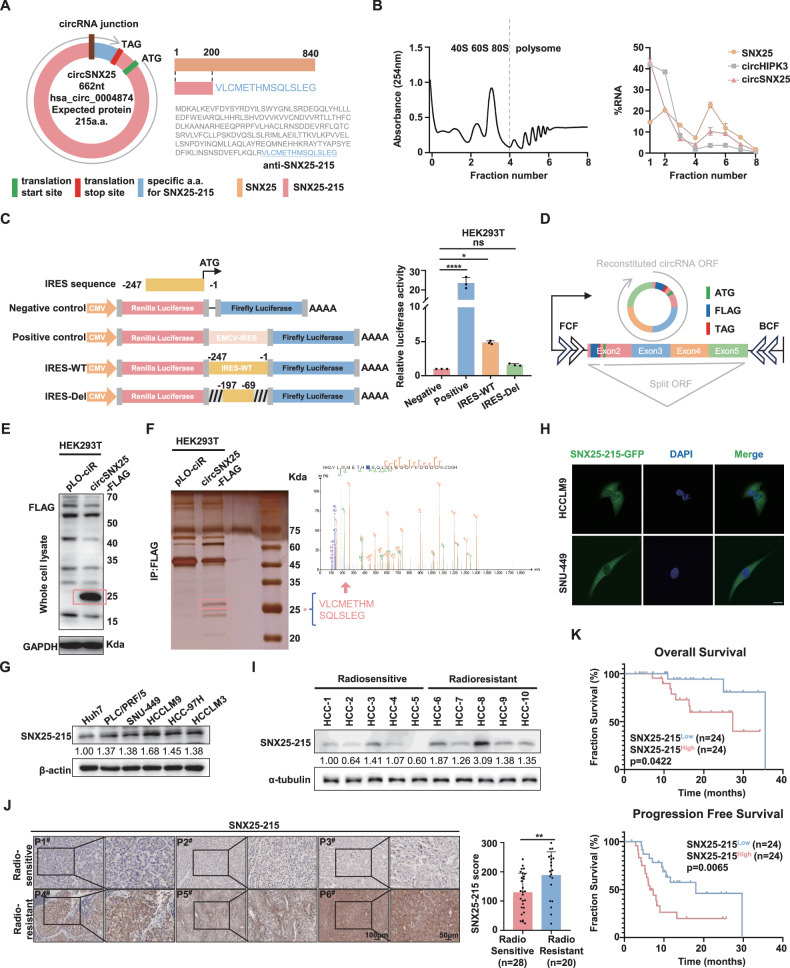
CircSNX25 encodes a novel protein SNX25-215. **A** Schematic of circSNX25 encoding SNX25-215. Positions of the start codon (ATG) and stop codon (TAG) are shown (left). The unique peptide sequence of SNX25-215, differing from the parental protein, is highlighted with blue (right). **B** Polysome profiling of HEK293T cells transfected with circSNX25 plasmid. qPCR analysis of circSNX25 in indicated fractions. CircHIPK3 and SNX25 were used as negative and positive controls, respectively. **C** Assessment of putative internal ribosome entry site (IRES) activity of circSNX25. IRES sequences and truncation mutants were cloned into the P-Luc2-IRES-Report vector. Encephalomyocarditis virus (EMCV) IRES served as a positive control, and an empty vector was the negative control (left). Relative luciferase activity was measured in HEK293T cells (right). **D** Schematic of the pLO-ciR-FLAG construct. Exons 2–5 of the Sorting Nexin-25 (SNX25) gene were cloned into a plasmid containing inverted repeats to promote circularization. A FLAG tag was inserted upstream of the stop codon. Start and stop codons are highlighted in green and red, respectively, with the FLAG coding sequence in blue. **E** Western blotting analysis of FLAG-tagged protein in HEK293T cells transfected with circSNX25-FLAG. GAPDH served as a loading control. **F** Immunoprecipitation (IP) and mass spectrometry (MS) analysis. Left: IP using an anti-FLAG antibody in HEK293T cells with circSNX25-FLAG overexpression. Right: Gel bands around 25 kDa were excised for MS analysis. **G** Western blotting (WB) analysis of SNX25-215 expression across various HCC cell lines. **H** Immunofluorescence (IF) analysis of SNX25-215 localization in HCCLM9 and SNU-449 cells. Green fluorescence indicates SNX25-215, while blue fluorescence marks the nucleus. Scale bar: 20 μm. **I** WB analysis of SNX25-215 expression in 5 radiosensitive and 5 radioresistant HCC tissues. **J** Immunohistochemical (IHC) staining of SNX25-215 in radiosensitive (n = 28) and radioresistant (n = 20) HCC samples. Staining indexes were calculated. Scale bar: 100 μm (left). Scale bar: 50 μm (right). **K** Kaplan–Meier analysis of progression-free survival (PFS) and overall survival (OS) in patients with high (n = 24) and low (n = 24) SNX25-215 expression in the Sun Yat-sen University First Affiliated Hospital (SYSUFAH) cohort. Quantitative data represent mean ± SD (n = 3 independent experiments) and statistical significance was assessed by one-way *ANOVA* with Dunnett’s multiple comparisons test (**C**), two-tailed unpaired Student’s *t* test (**J**) or log-rank test (**K**). (****p < 0.0001, **p < 0.01, *p < 0.05, ns: not significant).

Emerging evidence has indicated that some circRNAs possess protein-coding capacity [27]. As annotated in the circRNADb database, circSNX25 contains an open reading frame (ORF) of 648 nt that encodes a 215-amino acid protein. Translation initiates at the ATG start codon and terminates at the TAG stop codon after a single translation cycle. This putative protein, designated as SNX25-215, differs from the parental SNX25 protein by the presence of a unique 15-amino acid peptide located just after the back-splicing junction (Fig. 2A).

We then assessed the translational potential of circSNX25. The TransCirc database predicted that circSNX25 binds to polysomes, a hallmark of active translation [28]. To validate this, we transfected circSNX25 into HEK293T cells and performed sucrose density gradient centrifugation, followed by qPCR analysis of non-ribosomal, 40–80S ribosomal, and polysomal fractions. Similar to the linear SNX25 mRNA (positive control), circSNX25 was present in polysome fractions, whereas circHIPK3 (negative control) was predominantly distributed in the non-ribosomal fraction (Fig. 2B). These results suggest that circSNX25 is associated with polysomes, supporting its potential for translation. Since circRNAs lack a 5′ cap, translation initiation typically relies on internal ribosome entry sites (IRES) [29]. The CircBank database predicted a putative IRES sequence located 247 base pairs upstream of the circSNX25 ORF. To assess its activity, we cloned both the full-length and truncated versions of the IRES sequences into the P-Luc2-IRES-Report vector. The luciferase assay revealed that the full-length IRES effectively initiated translation, whereas the truncated version failed to activate (Fig. 2C). These findings confirm that circSNX25 meets the prerequisite for protein-coding potential.

To further confirm the translation of circSNX25, we constructed an expression vector pLO-ciR containing a FLAG tag sequence immediately upstream of the stop codon. This design ensures that a tagged protein would be produced only if a circular template is formed (Fig. 2D). In HEK293T cells, the pLO-ciR-FLAG produced a FLAG-tagged protein with the expected molecular weight, as predicted by the TransCirc database (Fig. 2E). To further validate this, immunoprecipitation (IP) was performed to enrich the FLAG-tagged protein, and a 15-amino acid peptide unique to the circSNX25 junction was identified by mass spectrometry (MS) (Fig. 2F).

To specifically detect SNX25-215, we generated an antibody targeting its unique 15-amino acid peptide sequence and validated its specificity for immunohistochemistry (IHC) (Fig. S2A). Using this antibody, we confirmed the endogenous expression of SNX25-215 across multiple HCC cell lines (Fig. 2G). Immunofluorescence (IF) staining revealed that SNX25-215, when expressed as a GFP-fused protein, was primarily localized in the cytoplasm of HCC cells (Fig. 2H). Additionally, IHC and Western blotting (WB) analyses demonstrated that SNX25-215 was significantly upregulated in HCC tissues compared to adjacent normal tissues (Fig. S2B, C). We further investigated the relationship between SNX25-215 expression and radiotherapy response in HCC patients. In 10 randomly selected HCC samples, the expression of SNX25-215 was significantly higher in radioresistant tissues compared to radiosensitive tissues, as confirmed by WB analysis (Fig. 2I). IHC staining of 48 pre-RT tumor biopsies from HCC patients at SYSUFAH revealed that SNX25-215 expression was significantly elevated in radioresistant samples (Fig. 2J). Based on IHC score analysis, patients were divided into high and low SNX25-215 expression groups using the median expression level as the cutoff. Kaplan-Meier analysis demonstrated that higher SNX25-215 levels were significantly correlated with shorter OS (p = 0.0422) and PFS (p = 0.0065) in patients receiving radiotherapy (Fig. 2K), suggesting that SNX25-215, encoded by circSNX25, could serve as a prognostic marker for HCC patients undergoing IR. Taken together, our results demonstrate that circSNX25 is translated into SNX25-215 in an IRES-dependent manner. SNX25-215 is endogenously expressed in both HCC cell lines and tissues, and its high expression correlates with poor prognosis in HCC patients receiving radiotherapy.

### SNX25-215 promotes radioresistance in HCC cells

**Fig. 3:**
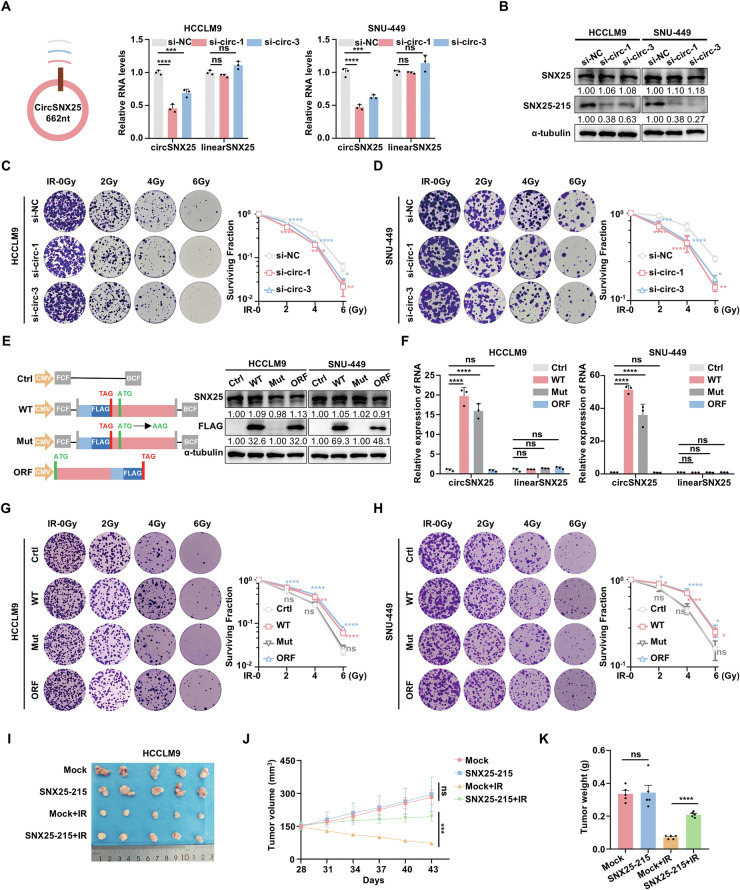
SNX25-215 promotes radioresistance in hepatocellular carcinoma Cells. **A** qPCR shows reduced circSNX25 and unchanged linear SNX25 RNA levels upon circSNX25 knockdown. **B** Western blotting confirms decreased SNX25-215 protein levels upon circSNX25 knockdown. **C**, **D** Colony formation assays reveal decreased surviving colonies after IR in HCCLM9 (**C**) and SNU-449 (**D**) cells with circSNX25 knockdown. **E** Schematic of four expression vectors: Ctrl (control), WT (FLAG-tagged circSNX25), MUT (FLAG-tagged circSNX25 with start codon mutation), and ORF (FLAG-tagged SNX25-215). Forward-circular frame (FCF) and backward-circular frame (BCF) sequences facilitate circRNA circularization. Western blotting analysis confirms FLAG-tagged SNX25-215 expression. **F** qPCR analysis of circSNX25 and linear SNX25 RNA expression levels in cells transfected with the four vectors. **G**, **H** Representative images and quantification of colony formation assays following IR treatment in HCCLM9 (**G**) and SNU-449 (**H**) cells transfected with the four vectors. **I**–**K** Xenograft study. HCCLM9 cells stably expressing either SNX25-215 or vector control were subcutaneously injected into NCG mice (n = 5 per group). Mice were treated with or without IR. Tumors were harvested and tumor volumes (**J**) and weights (**K**) were measured. Quantitative data represent mean ± SD (n = 3 independent experiments) and statistical analyses were performed using one-way *ANOVA* with Dunnett’s multiple comparisons test (**A**, **F**), two-tailed unpaired Student’s *t* tests (**J**, **K**) or two-way *ANOVA* with Dunnett’s multiple comparisons test (**C**, **D**, **G**, and **H**). (****p < 0.0001, ***p < 0.001, **p < 0.01, *p < 0.05, ns: not significant).

To investigate the functional role of circSNX25 in radiation resistance in HCC, we designed two independent short interfering RNAs (siRNAs) targeting the back-splicing junction site of circSNX25. These siRNAs effectively knocked down circSNX25 expression without affecting linear SNX25 levels in HCC cells (Fig. 3A, B). Clonogenic assays demonstrated that circSNX25 knockdown significantly reduced colony formation in HCCLM9 and SNU-449 cells following IR (Fig. 3C, D), while IR did not affect the expression of circSNX25 itself (Fig. S3A). Additionally, circSNX25 knockdown or overexpression did not significantly impact cell proliferation or metastasis in HCC, as demonstrated by Cell Counting Kit-8 (CCK-8) assay, transwell migration, and invasion assays (Fig. S3B–E).

To determine whether the observed effects were due to circSNX25 itself or its encoded protein SNX25-215, we established HCCLM9 and SNU-449 stable cell lines expressing one of four vectors: Ctrl (control), WT (FLAG-tagged circSNX25), MUT (FLAG-tagged circSNX25 with the ATG start codon mutation), and ORF (FLAG-tagged SNX25-215). The overexpression of circSNX25 and SNX25-215 was confirmed by qPCR and WB in all stable cell lines, while endogenous linear SNX25 levels remained unaltered (Fig. 3E, F). Clonogenic assays revealed that both circSNX25 and SNX25-215 enhanced radioresistance in HCC cells, whereas mutation of the ATG start codon, which prevents translation of SNX25-215, abolished this effect (Fig. 3G, H). These findings indicate that the radioresistant phenotype associated with circSNX25 is dependent on its ability to encode the SNX25-215 protein, as the radioresistant phenotype was lost when circSNX25 could not produce the protein SNX25-215.

HCCLM9 cells stably expressing Ctrl or ORF vectors were subcutaneously injected into NCG mice to validate these findings in vivo. Compared to the Ctrl group, SNX25-215-overexpressing tumors exhibited significantly larger volumes and weights following irradiation (Fig. 3I–K), demonstrating that SNX25-215 enhances radioresistance in HCC cells in vivo. Collectively, our results demonstrate that SNX25-215, rather than circSNX25 itself, enhances resistance to radiotherapy in HCC both in vitro and in vivo.

### SNX25-215 facilitates nuclear translocation of BAG6 by targeting GET4

**Fig. 4:**
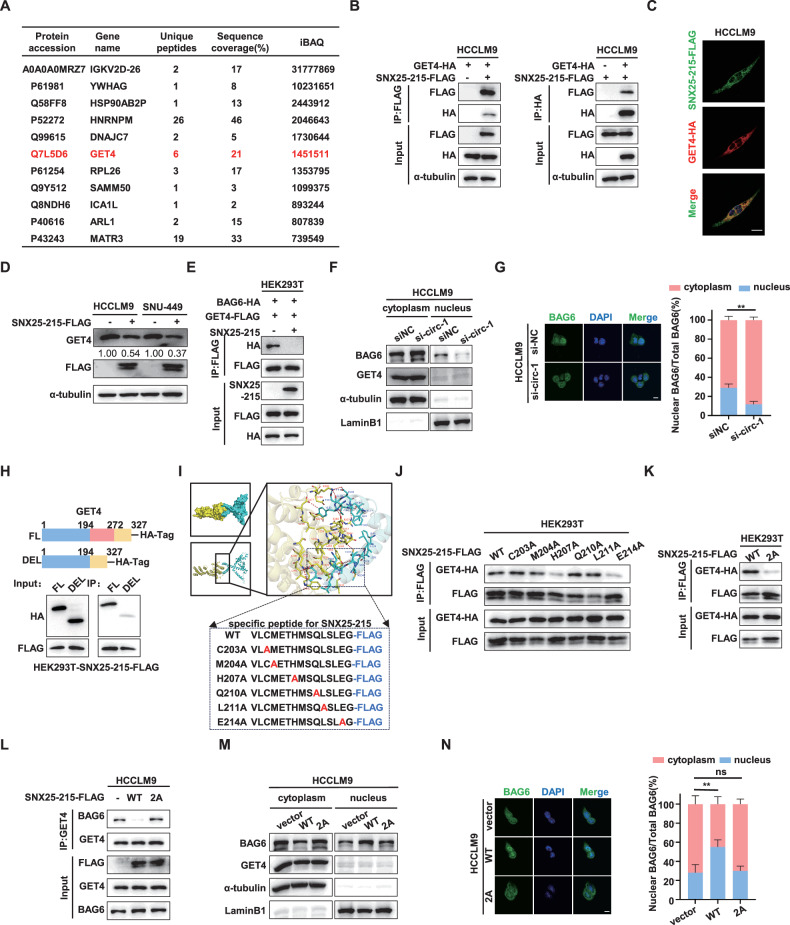
SNX25-215 facilitates nuclear translocation of BAG6 by targeting GET4. **A** Co-immunoprecipitation (Co-IP) using an anti-FLAG antibody in HCCLM9 cells overexpressing SNX25-215-FLAG, followed by mass spectrometry (MS) analysis, identified GET4 as a potential SNX25-215-interacting protein. **B** Co-IP confirmed the interaction between SNX25-215 and GET4 in HCCLM9 cells. **C** Immunofluorescence (IF) images showing co-localization of SNX25-215 with GET4 in HCCLM9 cells. Green fluorescence indicates SNX25-215, red fluorescence indicates GET4, and blue fluorescence marks the nucleus. Scale bar: 10 μm. **D** Western blotting (WB) analysis of GET4 levels in HCCLM9 and SNU-449 cells overexpressing SNX25-215. **E** Co-IP analysis demonstrating that SNX25-215 overexpression disrupts the interaction between BAG6 and GET4 in HEK293T cells. **F**, **G** WB (**F**) and IF (**G**) analysis assessed the cellular distribution of BAG6 in HCCLM9 after circSNX25 knockdown via siRNA. Green fluorescence indicates BAG6, and blue fluorescence indicates the nucleus. Scale bar: 10 μm. **H** Co-IP confirming that deletion of the BAG6-binding domain (residues 195–271) on GET4 reduces its binding to SNX25-215. Diagrams illustrate full-length and truncated GET4 constructs (upper panel). **I** Three-dimensional structural modeling of SNX25-215 and GET4. The overall binding mode is presented in a cartoon format (left), and a stick representation highlights residue interactions at the binding interface (middle). A zoomed-in view provides detailed insights into these interactions (right). Key interacting residues are highlighted, with mutant amino acids of SNX25-215 shown (lower panel). Yellow sticks represent interacting residues on GET4, and cyan sticks represent SNX25-215. Red dashed lines depict hydrogen bonds. Blue dashed lines depict salt bridges. Green dashed lines depict π-π stacking. **J** Co-IP verifying the interaction between mutant SNX25-215 (C203A, M204A, H207A, Q210A, L211A, E214A) and GET4 in HEK293T cells. **K** Co-IP in HEK293T cells demonstrated the interaction between SNX25-215 (WT or 2 A mutant) and GET4. The 2 A mutant contains E214A and H207A mutations. **L** Co-IP in HCCLM9 cells revealed the interaction between BAG6 and GET4, with the presence of SNX25-215 (WT or 2 A mutant). **M**, **N** WB (**M**) and IF (**N**) analysis examined the cellular distribution of BAG6 in HCCLM9 following the overexpression of SNX25-215 (WT or 2 A mutant). Green fluorescence indicates BAG6, and blue fluorescence indicates the nucleus. Scale bar: 10 μm. Quantitative data represent mean ± SD (n = 3 independent experiments) and statistical significance was determined by two-tailed unpaired Student’s *t* tests (G), one-way *ANOVA* with Dunnett’s multiple comparisons test (N). (**p < 0.01, ns: not significant).

To investigate the mechanism of SNX25-215-mediated radioresistance in HCC cells, we performed Co-IP using an anti-FLAG antibody in HCCLM9 cells overexpressing SNX25-215-FLAG, followed by MS analysis to identify potential SNX25-215-interacting proteins (Fig. S4A). GET4 was identified as a potential interacting partner of SNX25-215 (Figs. 4A and S4B). The interaction between SNX25-215-FLAG and GET4-HA was further confirmed by Co-IP in HCCLM9 cells (Fig. 4B). IF analysis demonstrated that exogenous SNX25-215-FLAG co-localized with GET4-HA in the cytoplasm (Fig. 4C). Interestingly, while SNX25-215 overexpression did not affect GET4 mRNA levels (Fig. S4C), it significantly reduced GET4 protein levels (Fig. 4D). These results suggest that SNX25-215 interacts with GET4 to promote GET4 protein degradation without affecting its transcription.

GET4 is a critical component of the cytosolic protein quality control complex known as the BAG6 complex, which maintains misfolded and hydrophobic patches-containing proteins in a soluble state and facilitates their proper delivery to the endoplasmic reticulum or directs them to the proteasome for degradation [30]. The stability of GET4 depends on its interaction with BAG6, which prevents GET4 from ubiquitylation and degradation [18, 21]. Meanwhile, GET4 functions as an intermolecular mask for the nuclear localization sequence (NLS) of BAG6, thereby limiting its nuclear translocation [31, 32]. This regulation is essential for DNA damage response (DDR) pathways [15]. Based on these findings, we hypothesized that SNX25-215 binding to GET4 disrupts the GET4/BAG6 interaction, thereby promoting GET4 degradation and the release of free BAG6, which subsequently translocates to the nucleus to enhance DDRs and promote radioresistance. Co-IP experiments revealed that SNX25-215 overexpression significantly reduced the interaction between GET4 and BAG6 (Fig. 4E), with no effect on the mRNA or protein levels of BAG6 (Fig. S4D, E). Furthermore, knockdown of circSNX25 significantly reduced the nuclear distribution of BAG6 (Figs. 4F, G and S5A, B). These results suggest that SNX25-215 competes with BAG6 for binding to GET4, leading to the dissociation of the BAG6/GET4 complex, which increases free BAG6 levels and enhances BAG6 nuclear translocation.

The region spanning residues 195–271 of GET4 is known to mediate its interaction with BAG6 [21]. We hypothesized that this region also serves as a critical binding site for SNX25-215. Deletion of this region in GET4 significantly diminished the interaction between SNX25-215 and GET4, suggesting that SNX25-215 competes with BAG6 for binding to this crucial region (Fig.4H). To further characterize the binding interface between SNX25-215 and GET4, we performed three-dimensional (3D) modeling and protein docking analysis (Fig. S6A, B). This analysis identified 20 residues on SNX25-215, 40% (8 of 20) of which are located within its unique 15-amino acid peptide sequence. Moreover, 23 interacting residues were identified on GET4, 90% (21 of 23) of which are situated within the 195-271 region. Notably, the binding site of SNX25-215’s unique peptide sequence on GET4 overlaps entirely with the GET4-BAG6 interaction site [21] (Fig. S6C). Among the identified residues, H207 and E214 on SNX25-215 were confirmed by Co-IP as critical for its interaction with GET4, while mutations at C203, M204, Q210, and L211 had no significant effect (Fig. 4I, J). Furthermore, double alanine substitution (2 A) at both H207 and E214 in SNX25-215 completely abolished its interaction with GET4 (Fig. 4K) and restored the GET4/BAG6 interaction in both HEK293T and HCCLM9 cells (Fig. 4L; S7A). These results suggest that SNX25-215 targets the BAG6-binding domain of GET4 through its unique 15-amino acid sequence, particularly residues H207 and E214, thereby competing with BAG6 for binding to GET4.

Based on the results shown in Fig. 4F, G, knockdown of SNX25-215 significantly reduced the nuclear translocation of BAG6. Further investigation revealed that overexpression of SNX25-215 significantly increased the nuclear distribution of BAG6, an effect that was abolished by the SNX25-215 mutation (Figs. 4M, N and S7B, C). Taken together, these results suggest that residues E214 and H207 within the unique 15-amino acid sequence of SNX25-215 competitively bind to GET4, disrupting the GET4/BAG6 complex and promoting the nuclear translocation of BAG6.

### SNX25-215 promotes DNA double-strand breaks repair in HCC cells by facilitating BAG6 nuclear translocation

Our previous studies demonstrated that SNX25-215 promotes the nuclear translocation of BAG6, a key regulator of DDRs. Abnormal activation of DDRs has been implicated to be responsible for radioresistance in cancer cells [13]. Notably, nuclear BAG6 interacts with the nucleoprotein p300 to facilitate p53 acetylation, a modification required for p53-mediated DDRs [16]. Additionally, BAG6 promotes H3K79 di-methylation, which is essential for the formation of 53BP1 foci in NHEJ pathway [17], and recruits BRCA1 to damage sites to enhance HR repair efficiency [18]. Furthermore, BAG6 stabilizes SET1A activity, increasing H3K4 di-methylation at the BRCA1 promoter and upregulating its expression [19]. Based on these findings, we hypothesized that SNX25-215 promotes BAG6 nuclear translocation, thereby promoting DNA damage repair and contributing to radioresistance in HCC.

**Fig. 5:**
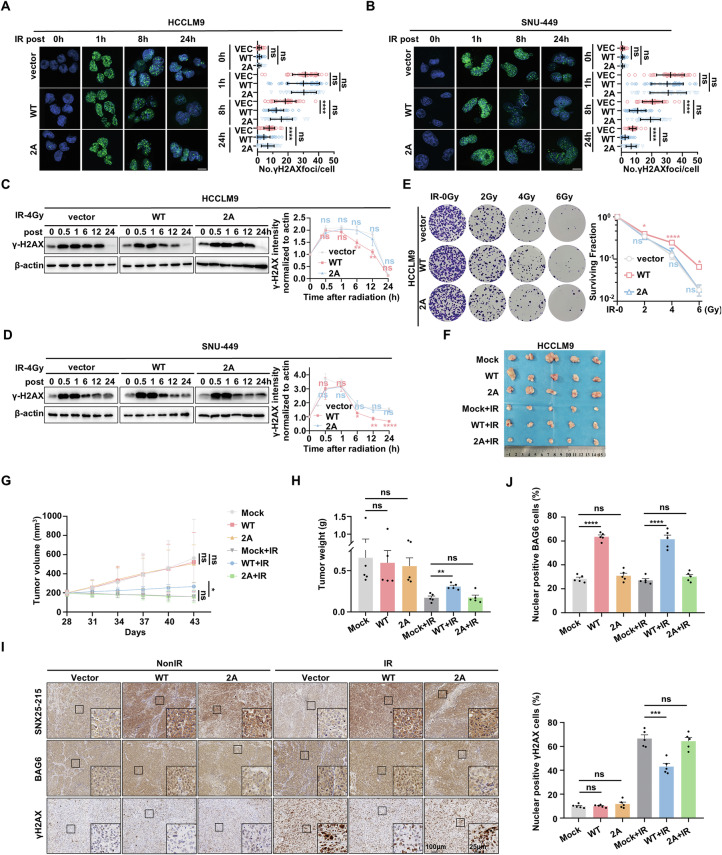
SNX25-215 promotes DNA double-strand breaks repair in hepatocellular carcinoma cells by facilitating BAG6 nuclear translocation. **A**, **B** Immunofluorescence (IF) assays showing phosphorylated H2AX (γ-H2AX) foci formation in HCCLM9 (**A**) and SNU-449 (**B**) cells transfected with either SNX25-215 (WT or 2 A) or vector control after ionizing radiation (IR) treatment. The 2 A mutant contains E214A and H207A mutations. Green fluorescence marks γ-H2AX, and blue fluorescence marks the nucleus. Left: Representative IF images; Right: Quantitative analysis of γ-H2AX foci from three independent experiments. Scale bar: 10 μm. **C**, **D** Western blotting analysis showing γ-H2AX levels in HCCLM9 (**C**) and SNU-449 (**D**) cells following the overexpression of SNX25-215 (WT or 2A mutant) at various time points post-IR treatment. Left: representative WB images; right: quantitative analysis (mean ± SD) from three independent experiments. **E** Colony formation assays evaluating the survival of HCCLM9 cells with stable overexpression of either wild-type SNX25-215-FLAG (WT) or the 2A mutant following different doses of irradiation. **F** HCCLM9 cells stably expressing different constructs were subcutaneously injected into NCG mice (n = 5 per group). Mice were then treated with or without IR at a dose of 4 Gy × 3 fractions. **G**, **H** Tumor volumes (**G**) and weights (**H**) were measured after sacrifice, demonstrating the effect of SNX25-215 on tumor growth and response to IR. **I** Representative images of paraffin sections from xenograft tumors stained with antibodies against SNX25-215, BAG6, and γ-H2AX. Scale bar: 100 μm (main), 25 μm (inset). **J** Quantification and statistical analysis of the nuclear localization rate of BAG6 and nuclear-positive γ-H2AX across all xenograft tumors. Quantitative data represent mean ± SD (n = 3 independent experiments) and statistical analyses were performed using one-way *ANOVA* with Dunnett’s multiple comparisons test or Kruskal–Wallis with Dunn’s multiple comparisons test (**A**–**D**, **G**, **H**, **J**, based on normality) and two-way ANOVA with Dunnett’s multiple comparisons test (**E**). (****p < 0.0001, ***p < 0.001, **p < 0.01, *p < 0.05, ns: not significant).

To investigate the role of SNX25-215 in DNA damage repair following IR, we assessed γ-H2AX foci formation and protein levels, a monitored marker in response to DNA DSBs [33]. While initial γ-H2AX levels did not differ between SNX25-215-overexpressing cells and controls at 1 h post-irradiation, SNX25-215-overexpressing cells exhibited a faster decrease in γ-H2AX levels over time, indicating enhanced DNA repair efficiency (Fig. 5A–D). In contrast, SNX25-215 knockdown led to a prolonged presence of γ-H2AX, suggesting impaired DNA repair capabilities (Fig. S8A–D). Furthermore, the accelerated repair observed in SNX25-215-overexpressing cells was abolished when a mutation was introduced in SNX25-215, highlighting its critical role in this process (Fig. 5A–D).

Functionally, SNX25-215 overexpression promoted radioresistance in HCCLM9 and SNU-449 cells, while the SNX25-215 mutant abolished this effect (Figs. 5E and S8E).

To validate these findings in vivo, we established a xenograft mouse model in NCG mice. Tumors derived from SNX25-215-overexpressing cells exhibited increased resistance to irradiation without affecting baseline tumor growth, as indicated by smaller reductions in tumor volume and growth rate following radiotherapy compared to controls. Importantly, this radioresistance was abolished in tumors expressing the SNX25-215 2 A mutant (Fig. 5F–H). These findings demonstrate that SNX25-215-mediated radioresistance in HCC relies on its interaction with GET4 to promote BAG6 nuclear translocation, as the SNX25-215 2 A mutation disrupts this interaction and restores radiosensitivity. Furthermore, IHC analysis of tumor sections showed increased nuclear localization of BAG6 due to SNX25-215 overexpression, an effect that was reversed by the SNX25-215 2 A mutant irrespective of irradiation. While IR exposure increased γ-H2AX foci and levels, SNX25-215 overexpression led to a reduction in these markers, an effect that was also reversed by the SNX25-215 2 A mutant (Fig. 5I, J).

Overall, these results demonstrate that SNX25-215 enhances DNA DSB repair and promotes radioresistance in HCC cells both in vitro and in vivo. These radioresistant properties can be reversed by the SNX25-215 2A mutant, which impairs the interaction between SNX25-215 and GET4, leading to BAG6 retention in the cytoplasm. Consequently, SNX25-215 promotes DNA DSB repair by targeting GET4, facilitating the dissociation of the GET4/BAG6 complex, and enhancing the nuclear translocation of BAG6.

### SNX25-215-mediated BAG6 nuclear translocation correlates with radiotherapy response in HCC patients

**Fig. 6:**
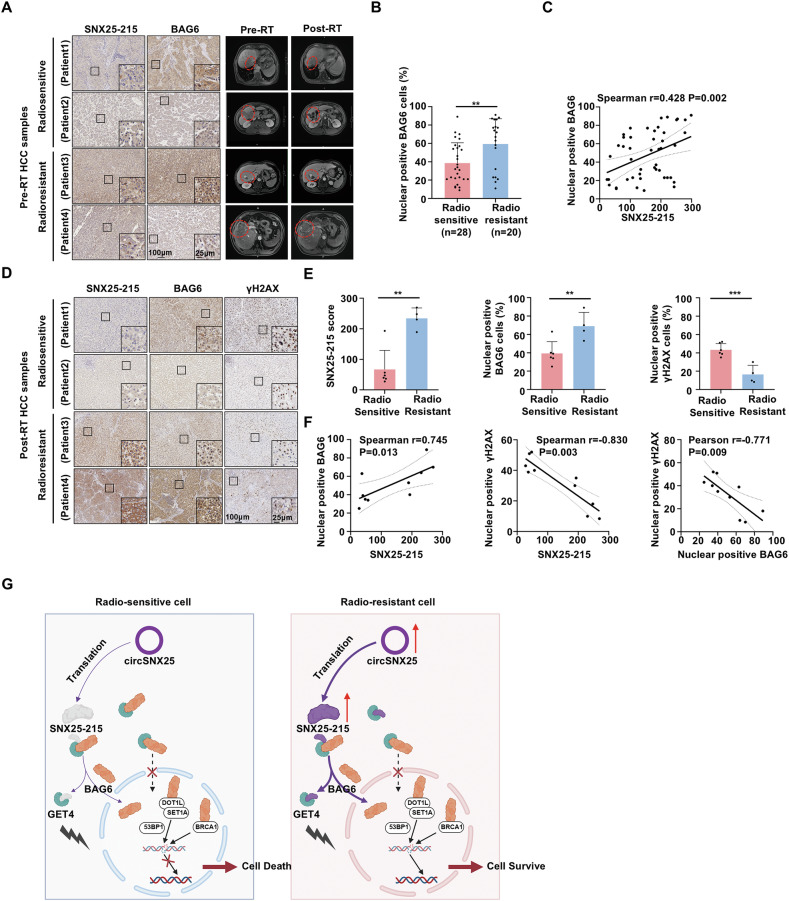
SNX25-215-mediated BAG6 nuclear translocation correlates with radiotherapy response in HCC patients. **A** Representative immunohistochemical (IHC) staining of indicated targets in tumor samples from hepatocellular carcinoma (HCC) patients who received surgical resection or liver biopsy before radiotherapy in the SYSUFAH cohort. Matched magnetic resonance imaging (MRI) scans from corresponding patients before and after radiotherapy (RT) are displayed. Scale bar: 100μm (main), 25 μm (inset). **B** Statistics of nuclear-positive BAG6 between radiosensitive (n = 28) and radioresistant (n = 20) HCC patients. **C** Correlation between nuclear-positive BAG6 and SNX25-215 in pre-RT HCC samples (n = 48). **D** Representative IHC staining of indicated targets in ten tumor samples from patients with HCC who received surgical resection or liver biopsy after radiotherapy. Scale bar: 100 μm (main), 25 μm (inset). **E** Statistics of indicated targets between radiosensitive (n = 6) and radioresistant (n = 4) post-RT HCC patients. **F** Pairwise correlations between SNX25-215, nuclear-positive BAG6 and nuclear-positive γ-H2AX in post-RT HCC samples (n = 10). **G** Working model depicting the mechanisms underlying SNX25-215-mediated radioresistance in HCC. SNX25-215, encoded by circSNX25, competes with BAG6 for binding to GET4, leading to BAG6 nuclear translocation. In the nucleus, BAG6 enhances DNA damage repair, thereby promoting radioresistance in HCC. Created in BioRender. shuping, l. (2025) https://BioRender.com/ii62kdo. Statistical analyses were performed using two-tailed unpaired Student’s *t* tests (**B**, **E**; ***p < 0.001, **p < 0.01) or correlation analyses (Spearman’s or Pearson’s tests based on normality testing; **C**, **F**).

To investigate the clinical relevance of SNX25-215-mediated BAG6 nuclear translocation, we performed IHC staining on 48 pre-radiotherapy (pre-RT) HCC tissue samples. The analysis revealed that the nuclear translocation of BAG6 was significantly higher in radioresistant HCC samples compared to radiosensitive ones (Fig. 6A, B). Moreover, BAG6 nuclear translocation was positively correlated with SNX25-215 expression (r = 0.428, p = 0.002) (Fig. 6C). These findings suggest that SNX25-215-mediated BAG6 nuclear translocation may play a crucial role in conferring radioresistance in HCC.

To further validate these findings, we analyzed biopsy samples from 10 HCC patients collected post-radiotherapy. Consistent with the pre-RT data, both SNX25-215 expression and nuclear-localized BAG6 levels were significantly elevated in radioresistant samples and exhibited a strong positive correlation (r = 0.745, p = 0.013). Notably, nuclear γ-H2AX levels were significantly lower in radioresistant samples and negatively correlated with SNX25-215 expression (r = −0.830, p = 0.003). Similarly, nuclear γ-H2AX levels were inversely correlated with nuclear-localized BAG6 (r = −0.771, p = 0.009) (Fig. 6D–F).

Taken together, these clinical data highlight the pivotal role of SNX25-215 and BAG6 nuclear translocation in enhancing DNA DSB repair, ultimately contributing to radioresistance in HCC patients (Fig. 6G).

## Discussion

CircRNAs have emerged as important players in cancer biology due to their high stability and diverse functional roles, including their recently discovered protein-coding potential [26, 34]. These circRNA-encoded proteins can either have C-terminal extensions or be novel proteins generated by ORF shifts caused by non-canonical translation initiation mechanisms [27]. CircRNA-encoded proteins function in two distinct ways. Firstly, they can act as decoys or competitors, binding to molecules that would typically interact with their corresponding linear protein isoforms. This interaction can protect the full-length proteins from degradation and sequestration, indirectly inhibiting the function of their full-length counterparts [35]. For example, SHPRH-146aa protects SHPRH protein from ubiquitin-mediated degradation, thereby acting as a tumor suppressor by targeting PCNA for degradation [36]. Secondly, circRNA-encoded proteins have independent functions distinct from those of their cognate genes [35]. For example, E-cadherin protein variant encoded by a circular E-cadherin RNA enhances epidermal growth factor receptor (EGFR)-signal transducer and activator of transcription 3 (STAT3) signaling in glioblastoma to promote stemness of glioblastoma stem cells [37]. In this study, we identified circSNX25 as an upregulated circRNA in radioresistant HCC, encoding a novel protein, SNX25-215. SNX25-215 is derived from the second to fifth exons of the SNX25 gene, with an additional 15 amino acids at the C terminus beyond the back-splicing junction. Although SNX25-215 shares the first 200-amino-acid sequence with its cognate SNX25, our findings suggest that it does not function as a decoy or competitor. Instead, the addition of the 15-amino-acid sequence provides distinct biological functions that promote radioresistance in HCC.

Further mechanistic studies revealed that SNX25-215, as a novel protein, competes with BAG6 for binding to GET4 via two key amino acid sites within its unique peptide sequence. This competition disrupts the GET4/BAG6 complex, exposing BAG6’s NLS and facilitating its translocation into the nucleus, where it regulates the DNA damage responses [15]. Specifically, nuclear BAG6 recruits critical DNA repair proteins including BRCA1 and 53BP1 to damage sites, promoting DNA repair efficiency [17, 18]. Additionally, BAG6 interacts with the nucleoprotein p300 to facilitate p53 acetylation, a critical step in DNA damage response [16]. BAG6 also modulates chromatin structure by interacting with histone methyltransferases, such as DOT1L and SET1A, to promote H3K79 and H3K4 di-methylation, respectively. These modifications are essential for the formation of 53BP1 foci and the upregulation of BRCA1 expression, establishing a feedback loop that further enhances DNA repair [17, 19]. Together, these protective DNA damage responses contribute to tumor resistance to radiation-induced DNA damage. The ability of tumor cells to repair radiation-induced DNA damage is a key determinant of radiotherapy efficacy, and abnormal activation of DNA damage response pathways has been implicated in radioresistance, posing a significant challenge in cancer treatment [38, 39]. Collectively, our findings demonstrate that two key sites within the unique peptide sequence of SNX25-215 modulate the cellular distribution of BAG6, thereby enhancing DNA repair and promoting radioresistance in HCC.

Our clinical data revealed that circSNX25, its protein product SNX25-215, and BAG6 nuclear translocation are significantly associated with radiotherapy resistance in HCC patients. In pre-RT cohorts, both circSNX25 and SNX25-215 were upregulated in radioresistant patients and correlated with shorter OS and PFS, highlighting their potential as prognostic biomarkers. Post-RT cohorts further confirmed that SNX25-215-mediated BAG6 nuclear translocation promotes radiotherapy resistance by enhancing DNA damage repair, solidifying their potential as therapeutic targets for overcoming radioresistance in HCC. Despite these promising findings, our study has several limitations, including a relatively small sample size and lack of longitudinal patient data. Larger clinical cohorts are necessary to validate circSNX25 and SNX25-215 as reliable biomarkers. Additionally, while xenograft models provided valuable insights, more complex models that better replicate the human tumor microenvironment are essential for evaluating therapeutic strategies. Future research should focus on developing targeted interventions, such as gene silencing of circSNX25, monoclonal antibodies, or small molecule inhibitors targeting the unique peptide sequence of SNX25-215, to overcome radiotherapy resistance in HCC.

In conclusion, our study identifies circSNX25 as a novel protein-coding circRNA that is significantly upregulated in radioresistant HCC patients and strongly correlates with poor prognosis. Mechanistically, circSNX25 encodes a functional protein, SNX25-215, which disrupts the GET4/BAG6 complex by competitively binding to GET4. This disruption facilitates BAG6 nuclear translocation, leading to enhanced DNA repair capacity and ultimately conferring radiation resistance in HCC. These findings highlight circSNX25 and its encoded protein SNX25-215 as promising predictive biomarkers and therapeutic targets, providing a potential avenue to overcome radioresistance and improve radiotherapy efficacy in HCC patients.

## Supplementary information

Supplementary materials

Original Western blots

## Data Availability

The sequencing data were deposited in the NCBI database under the accession ID SRP527697. The remaining data are available within the Article, Supplementary Information.
