# Supplementary material for: Circular SNX25 encoded radioresistance augmenter facilitates DNA damage repair in hepatocellular carcinoma by targeting BAG6-GET4 interaction
Source: Cell Death Dis. 2025 Oct 21;16(1):734. doi: 10.1038/s41419-025-08026-9 (PMC12541074; doi:10.1038/s41419-025-08026-9)
Supplement: Supplementary file 1 — Supplementary materials [file 41419_2025_8026_MOESM1_ESM.pdf]

Supplementary Figures

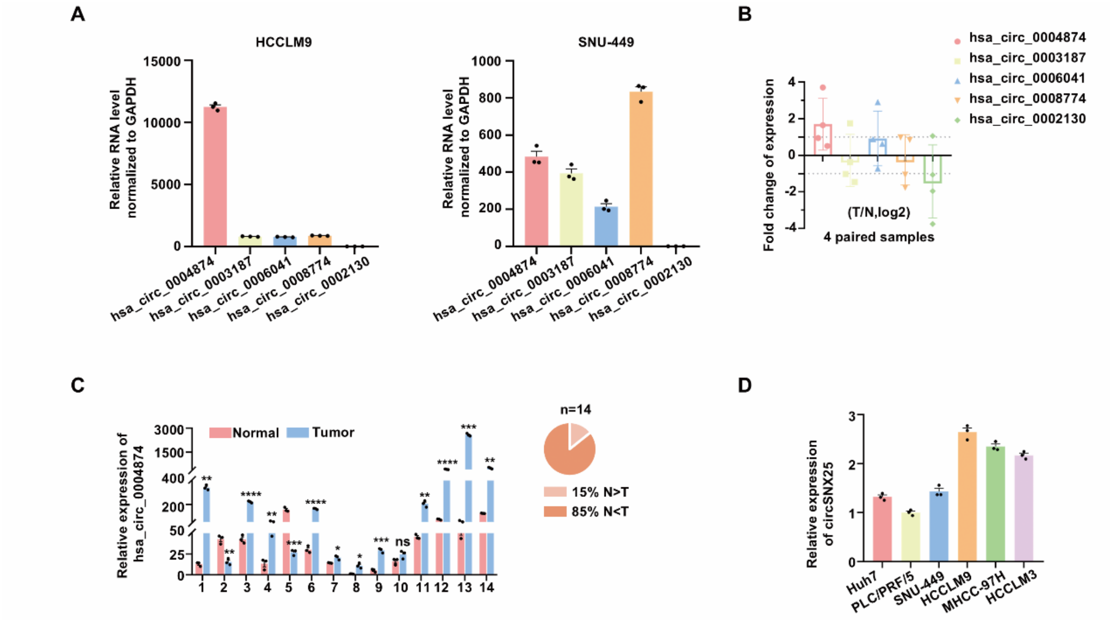

Figure S1. Screening of differentially expressed circRNAs in radiation-resistant HCC

A. Validation of 5 candidate circRNAs by quantitative polymerase chain reaction (qPCR) in HCCLM9 and SNU-449 cell lines.

B. Relative expression levels of the 5 circRNAs in four paired HCC and normal tissues.

C. Expression of hsa_circ_0004874 in 14 paired HCC and normal tissues (left), and the ratio of hsa_circ_0004874 expression in these 14 samples (right).

D. Relative expression of circSNX25 in a panel of HCC cell lines.

Quantitative data represent mean ± SD (n = 3 independent experiments) and statistical analyses were performed using two-tailed unpaired Student's *t* tests (C). (*****p* < 0.0001, ****p* < 0.001, ***p* < 0.01, **p* < 0.05, ns: not significant)

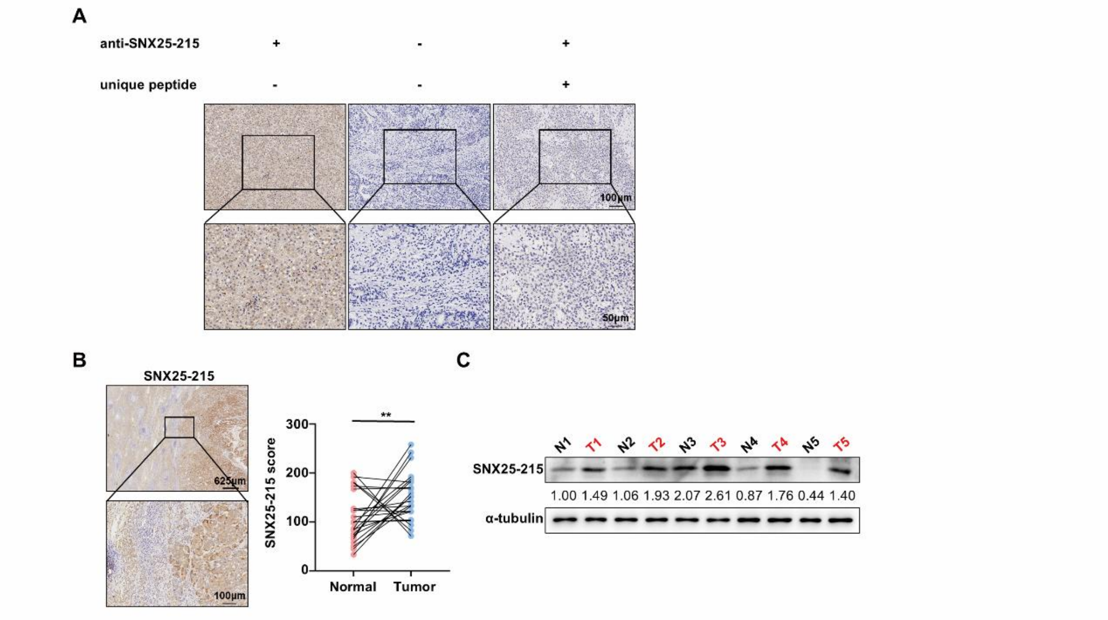

Figure S2. CircSNX25 encodes a novel protein SNX25-215

A. Peptide blocking experiment demonstrating the specificity of the SNX25-215 antibody for immunohistochemistry (IHC). Scale bar: 100 μm (upper); Scale bar: 50 μm (lower).

B. IHC analysis of SNX25-215 in 24 pairs of HCC and adjacent tissues: Representative images (left) and paired staining indexes (right). Scale bar: 625 μm (upper); Scale bar: 100 μm (lower).

C. Western blotting (WB) analysis of SNX25-215 expression in 5 pairs of HCC and adjacent tissues.

Statistical analyses were performed using two-tailed paired Student's *t* tests (B). (** $p < 0.01$)

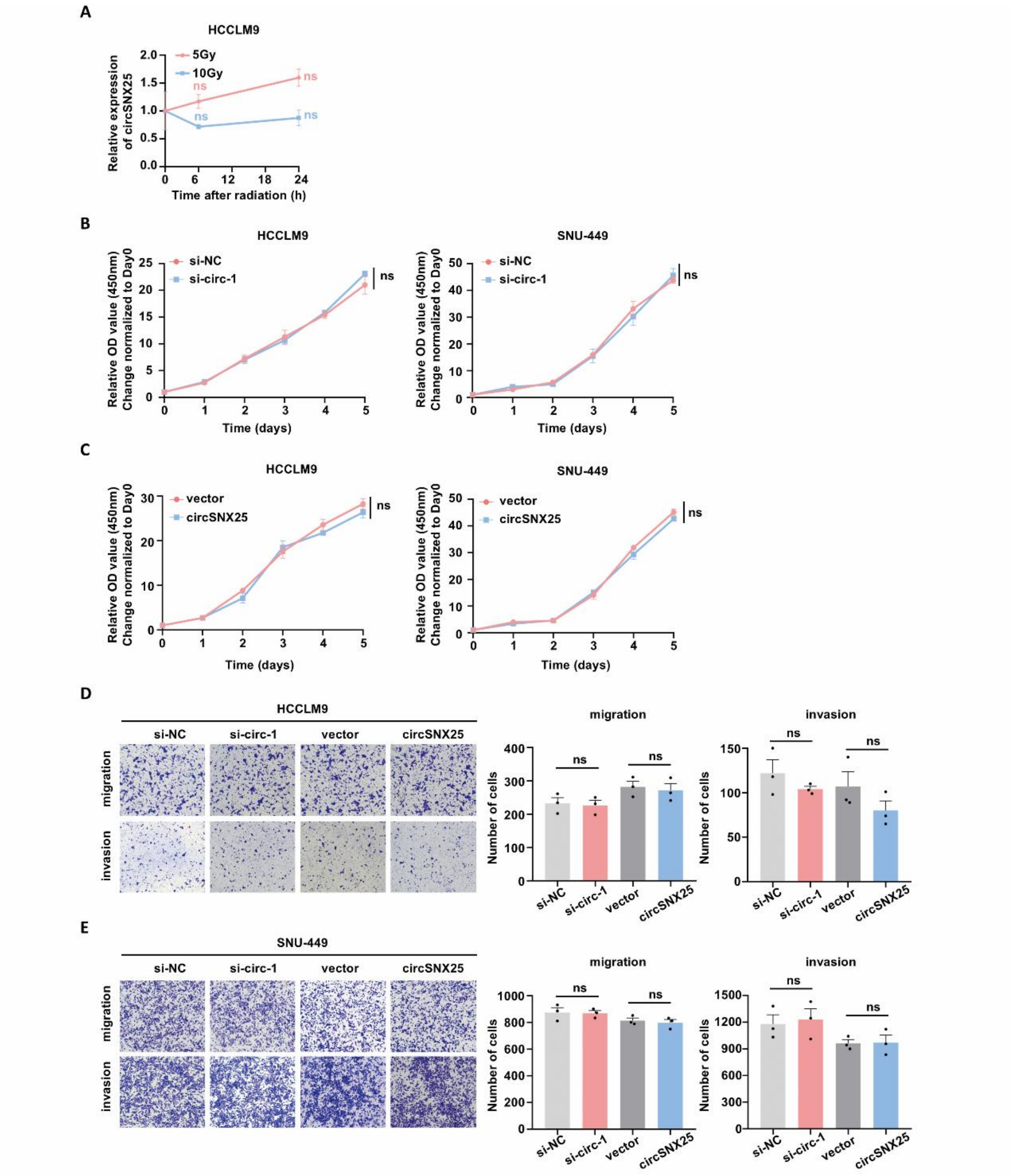

Figure S3. circSNX25 does not affect HCC cell proliferation, migration, or invasion

A. qPCR analysis of circSNX25 expression levels under ionizing radiation (IR).

B-C. Cell Counting Kit-8 (CCK-8) assays showing no significant difference in proliferation between HCCLM9 and SNU-449 cells with circSNX25 knockdown (B) or overexpression (C).

D-E. Transwell assays demonstrating no significant change in migration and invasion capabilities of HCCLM9 (D) and SNU-449 (E) cells upon circSNX25 overexpression or knockdown.

Quantitative data represent mean ± SD (n = 3 independent experiments) and statistical analyses were performed using one-way *ANOVA* with Dunnett's multiple comparisons test (A), two-tailed unpaired Student's *t* tests (B-E). (ns: not significant)

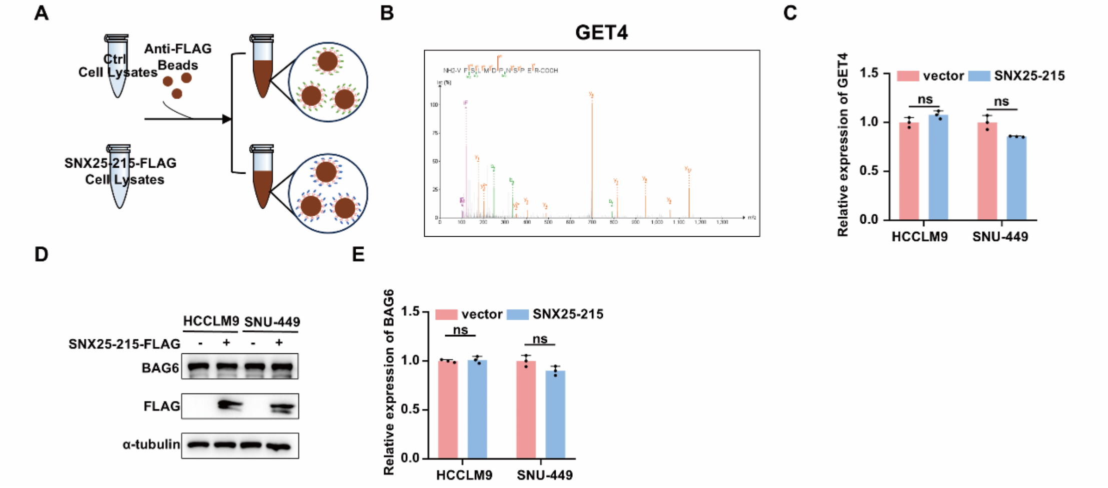

Figure S4. Validation of GET4 interactions with SNX25-215

A. Flow Chart of co-immunoprecipitation (Co-IP) and mass spectrometry (MS) assays in HCCLM9 cells overexpressing SNX25-215-FLAG. A FLAG antibody was utilized to enrich SNX25-215-FLAG and its interacting proteins.

B. Peptides recovered from GET4, identified by MS.

C. qPCR analyses assessing GET4 mRNA expression in HCCLM9 and SNU-449 cells overexpressing SNX25-215.

D. Western blotting (WB) analysis of BAG6 levels in HCCLM9 and SNU-449 cells overexpressing SNX25-215.

E. qPCR analyses assessing BAG6 mRNA expression in HCCLM9 and SNU-449 cells overexpressing SNX25-215.

Quantitative data represent mean ± SD (n = 3 independent experiments) and statistical analyses were performed using two-tailed unpaired Student's *t* tests (C, E). (ns: not significant)

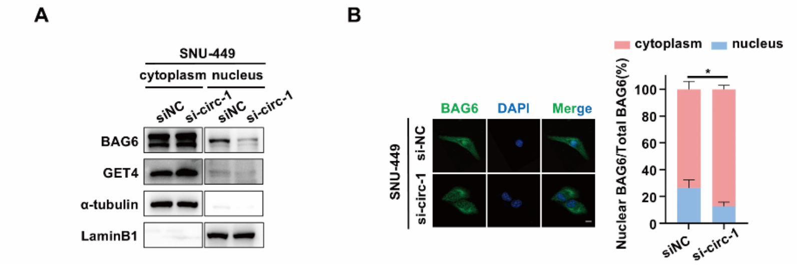

Figure S5. SNX25-215 knockdown inhibits BAG6 nuclear translocation.

A-B. WB analysis (A) and immunofluorescence (IF) (B) analysis showing the subcellular distribution of BAG6 in SNU-449 cells following transfection with circSNX25 siRNA or control siRNA. Green fluorescence indicates BAG6, and blue fluorescence indicates the nucleus. Scale bar: 10 µm.

Quantitative data represent mean ± SD (n = 3 independent experiments) and statistical analyses were performed using two-tailed unpaired Student's *t* tests (B). (**p* < 0.05)

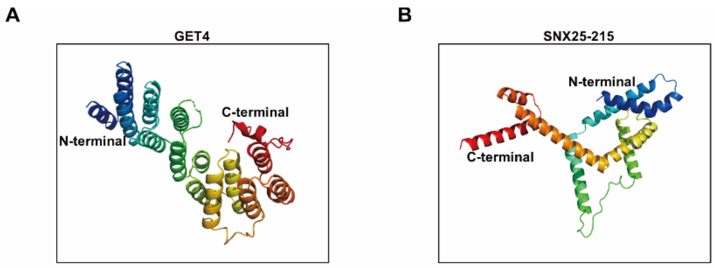

C

The contact list between GET4 with SNX25-215

ChainA	Residue	ChainB	Residue	Energy	Interaction type
GET4	Lys251(NZ)	SNX25-215	Glu214(O, OE1, OE2)	-30.45	Hydrogen bond, salt bridge, VDW
GET4	Lys289(NZ)	SNX25-215	Glu181(OE1, OE2)	-25.46	Hydrogen bond, salt bridge, VDW
GET4	Lys289(NZ)	SNX25-215	Asp182(OD1, OD2)	-15.71	Hydrogen bond, salt bridge, VDW
GET4	Glu260(OE2)	SNX25-215	Tyr180(N)	-5.15	Hydrogen bond, VDW
GET4	Arg267(NH2)	SNX25-215	Asn188(OD1)	-5.02	Hydrogen bond, VDW
GET4	Lys289(NZ)	SNX25-215	Ser179(OG)	-4.9	Hydrogen bond, VDW
GET4	Phe242(Benzene ring)	SNX25-215	His207(Imidazole ring)	-3.14	π-π stacking, VDW
GET4	Glu260(OE2)	SNX25-215	Ser179(CA)	-2.3	Hydrogen bond, VDW
GET4	Asn273(O)	SNX25-215	Tyr180(OH)	-1.72	Hydrogen bond, VDW
GET4	Phe242	SNX25-215	Leu211	-1.43	VDW
GET4	Gln261(O)	SNX25-215	Cys203(SG)	-1.3	Hydrogen bond, VDW
GET4	Leu245(CB)	SNX25-215	Glu214(OE2)	-0.99	Hydrogen bond, VDW
GET4	Tyr262(OH)	SNX25-215	Cys203(O)	-0.89	Hydrogen bond, VDW
GET4	Gln290	SNX25-215	Glu181	-0.74	VDW
GET4	Asp277(OD1)	SNX25-215	Tyr180(CE1)	-0.65	Hydrogen bond, VDW
GET4	Glu260	SNX25-215	Phe183	-0.6	VDW
GET4	Trp241	SNX25-215	Leu211	-0.59	VDW
GET4	Val254	SNX25-215	Gln210	-0.57	VDW
GET4	Arg267	SNX25-215	Ile184	-0.55	VDW
GET4	Asn238	SNX25-215	His207	-0.53	VDW
GET4	Arg267	SNX25-215	Ile187	-0.53	VDW
GET4	Pro264	SNX25-215	Leu199	-0.46	VDW
GET4	Phe242	SNX25-215	Gln210	-0.44	VDW
GET4	Val254	SNX25-215	Glu214	-0.43	VDW
GET4	Leu276	SNX25-215	Tyr180	-0.43	VDW
GET4	Asn273	SNX25-215	Ile184	-0.3	VDW
GET4	Gln261	SNX25-215	Thr206	-0.29	VDW
GET4	Phe242	SNX25-215	Glu214	-0.28	VDW
GET4	Gln261	SNX25-215	Leu202	-0.16	VDW
GET4	Gln263	SNX25-215	Ile187	-0.16	VDW
GET4	Gln290	SNX25-215	Tyr180	-0.15	VDW
GET4	Gln261	SNX25-215	Leu199	-0.13	VDW
GET4	Phe239	SNX25-215	His207	-0.08	VDW
GET4	Val257	SNX25-215	Gln210	-0.08	VDW
GET4	Ala246	SNX25-215	Glu214	-0.03	VDW
GET4	Val233	SNX25-215	Arg200	-0.02	VDW
GET4	Glu260	SNX25-215	Ala177	-0.02	VDW
GET4	Leu258	SNX25-215	His207	-0.01	VDW
GET4	Tyr262	SNX25-215	Met204	0.03	VDW
GET4	Gln263	SNX25-215	Ile184	0.15	VDW
GET4	Arg267	SNX25-215	Leu196	0.17	VDW
GET4	Tyr262	SNX25-215	His207	0.32	VDW
GET4	Lys289	SNX25-215	Tyr180	0.34	VDW
GET4	Cys259	SNX25-215	Tyr180	0.62	VDW
GET4	Gln263	SNX25-215	Tyr180	0.68	VDW
GET4	Pro235	SNX25-215	Arg200	0.98	VDW
GET4	Leu258	SNX25-215	Gln210	1.5	VDW

Figure S6. Structural modeling and protein docking of SNX25-215 and GET4

A-B. Predicted three-dimensional (3D) model of GET4 (A) and SNX25-215 (B).

C. Interaction analysis between GET4 and SNX25-215. Residues in GET4 involved in binding to the BAG6 are highlighted in red. Residues within the SNX25-215 unique peptide are highlighted in blue.

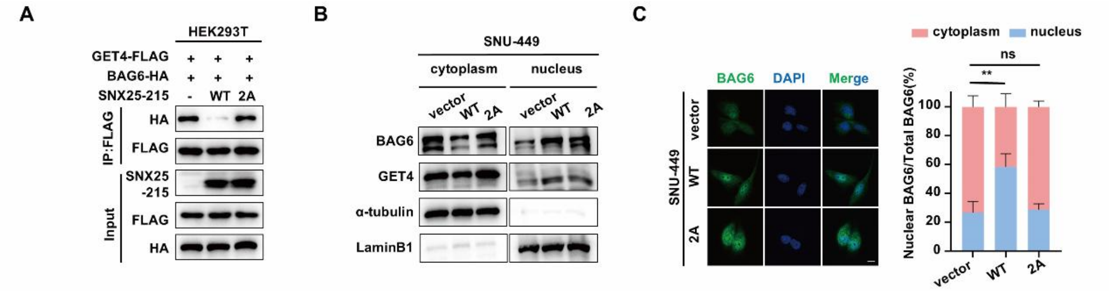

Figure S7. SNX25-215 facilitates nuclear translocation of BAG6

A. Co-IP in HEK293T cells reveals the interaction between BAG6 and GET4, with the presence of SNX25-215 (WT or 2A mutant). The 2A mutant contains E214A and H207A mutations.

B-C. WB (B) and IF (C) assays assessing the subcellular distribution of BAG6 in SNU-449 cells transfected with either SNX25-215 (WT or 2A) or vector control. Green fluorescence indicates BAG6, and blue fluorescence indicates the nucleus. Scale bar: 10 μm.

Quantitative data represent mean ± SD (n = 3 independent experiments) and statistical analyses were performed using one-way *ANOVA* with Dunnett's multiple comparisons test (C). (**$p < 0.01$, ns: not significant)

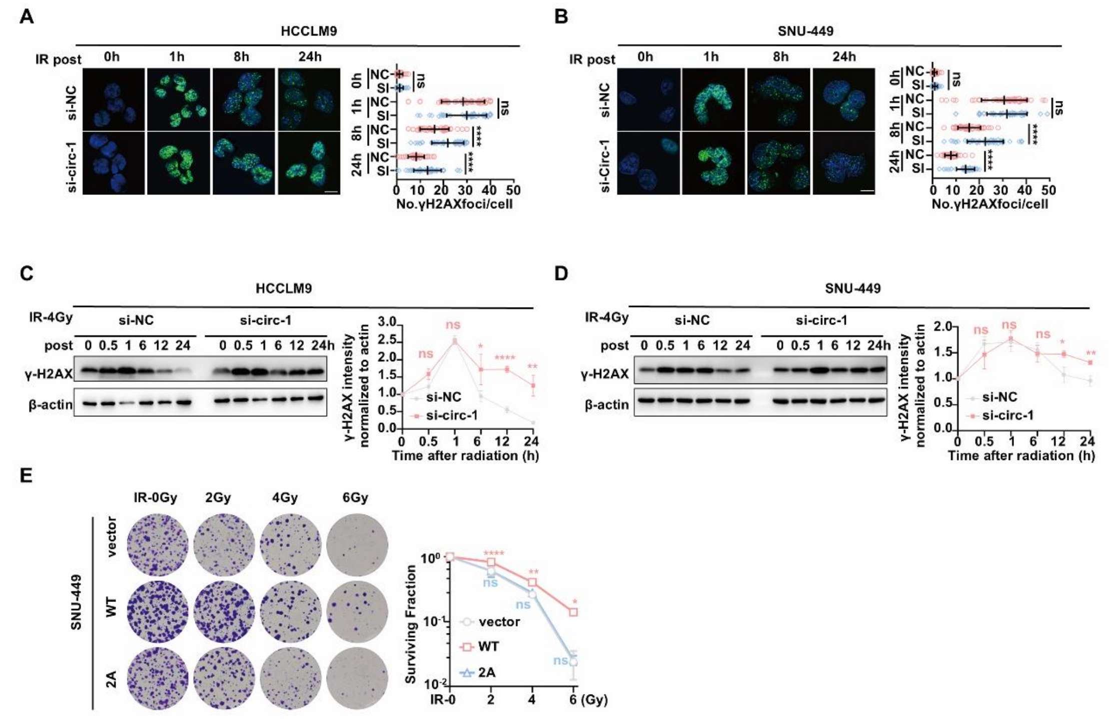

Figure S8. SNX25-215 promotes DNA double-strand breaks repair in hepatocellular carcinoma cells by facilitating BAG6 nuclear translocation.

A-B. IF analysis showing γ-H2AX foci in HCCLM9 (A) and SNU-449 (B) cells following transfection with circSNX25 siRNA or control siRNA at specified time points after irradiation (IR). Left: Representative IF images; Right: Quantitative analysis of γ-H2AX foci from three independent experiments. Scale bar: 10 μm.

C-D. WB analysis of γ-H2AX levels in HCCLM9 (C) and SNU-449 (D) cells with circSNX25 knockdown at various time points post-IR treatment. Representative WB images (left) and quantitative analysis (right) are presented as the mean ± SD from three independent experiments.

E. Colony formation assay evaluating the survival of SNU-449 cells exposed to different IR doses with stable overexpression of either SNX25-215-FLAG (WT or 2A). The 2A mutant contains E214A and H207A mutations. Representative images (left) and cell survival curves (right) are presented as the mean ± SD from three independent experiments.

Quantitative data represent mean ± SD (n = 3 independent experiments) and statistical analyses were performed using two-tailed unpaired Student's *t* tests (A-D) or two-way

ANOVA with Dunnett's multiple comparisons test (E). (****$p < 0.001$, **$p < 0.01$, *$p < 0.05$, ns: not significant).

Table S1. Primers for qPCR

Gene	Forward	Reverse
U6	CGCTTCGGCAGCACATATAC	TTCACGAATTTGCGTGTCAT
U3	TTCTCTGAGCGTGTAGAGCACCGA	GATCATCAATGGCTGACGGCAGTT
GAPDH	CTTCATTGACCTCAACTACATG	CTCGCTCCTGGAAGATGGTGA
SNX25	CCTACACCTATGCCCCCTCT	CAGCTTTCATTGCCGCAGTT
circHIPK3	ATGTTGGTGGATCCTGTTCG	GGGTAGACCAAGACTTGTGA
GET4	CAAAAGTAGCGCATCGGTGG	TGTCCTATGCGGTCGAGGTA
BAG6	CTGGTCATGGCCAAACCCT	ATTGGTACCCAGACCGGC
ACTB	GGGAAATCGTGCGTGACATTAAG	TGTGTTGGCGTACAGGTCTTTG
hsa_circ_0007619	AGAGAGTCAAGAAAGACCTGGA	GCCTTGTTGGATTTCTTTGTCT
hsa_circ_0006260	TGCCCTTTTTGCTAGCTGGT	AACCGCACAAACACTGGTTTA
hsa_circ_0004874	CCTACACCTATGCCCCCTCT	TCGAACACTTCTTTCAGAGCTT
hsa_circ_0003731	GAAGAAAGCGTCTCCTGATGG	TCAGAAATGGAGTGCGTTGTC
hsa_circ_0008774	CACCAGGATCTCACCATGCAA	GGCGGGTTTTCCCATACTCC
hsa_circ_0000019	ACATAATGAGACTGGTGGACCG	AACTTTCCGGAGCCTCTTCC
hsa_circ_0001438	ATGGTCCTGGTGAAAACGTC	GCCTTGTTGGATTTCTTTGTCT
hsa_circ_0002130	TTGAAAGACTCCATCACCACG	CTCTGGGAACTCACTTCGGG
hsa_circ_0017586	GGTACCTATATGCTGAATAAACCCA	TGCACTCTCTCCTCCGTAGT
hsa_circ_0006041	AGTGATTGCTCCTATGCTTGC	GTTCAGGCCATGGACAGTCT
hsa_circ_0003187	TTCCGAGCTCCTCTTTCAACT	CATGGGAGTCCTGGATGTCAA
hsa_circ_0003793	GGAAAGCCCCTCAAACCATT	GCAGACAGTAGCCAAATCACAA

Table S2. Resources table

REAGENT or RESOURCE	SOURCE	IDENTIFIER
**Antibodies**		
SNX25-215 (WB 1:1000, IHC 1:100)	DIA-AN	C3703
SNX25 (WB 1:500)	Invitrogen	PA5-78595
FLAG (WB 1:1000, IF 1:200)	Sigma-Aldrich	F1804
FLAG (WB 1:1000)	Proteintech	20543-1-AP
HA (WB 1:5000, IF 1:400)	CST	#3724
γH2AX (WB 1:1000, IHC 1:1000, IF 1:400)	CST	#2577
BAG6 (WB 1:1000, IHC 1:2000, IF 1:50)	Proteintech	26417-1-AP
GET4 (WB 1:1000)	Proteintech	27768-1-AP
Lamin B1 (WB 1:1000)	Proteintech	12987-1-AP
GAPDH (WB 1:1000)	Proteintech	10494-1-AP
Alpha Tubulin (WB 1:1000)	Proteintech	11224-1-AP
Beta Actin (WB 1:1000)	Proteintech	20536-1-AP
**Chemicals**		
DMEM	Corning	10-013-CVRC
Diaminobenzidine (DAB)	Dako	K406511-2
GoScript Reverse Transcription System	Promega	A5001
RealUniversal Color PreMix(SYBR Green)	TIANGEN	FP201-02
Cell lysis buffer	CST	9803S
DAPI	Beyotime	C1006
TRIzol	Invitrogen	15596026
Lipofectamine3000	Invitrogen	L3000015
Anti-FLAG® M2 Magnetic Beads	Sigma-Aldric	M8823
Lipofectamine RNAiMAX	Invitrogen	13778150
RNase R	Geneseed	R0301
**Critical Commercial Assays**		
Cell Counting Kit-8	Dojindo, Kumamoto, Japan	Cat#CK04
Pierce BCA Protein Assay Kit	Thermo Fisher	23227
Minute™ Cytoplasmic and Nuclear Extraction Kits	Invent	SC-003
SuperSignal West Pico Chemiluminescent Substrate	Thermo Fisher	RB231022
Dig RNA labeling kit (SP6 T7)	Roche	11175025910
Pierce Crosslink Magnetic IP/Co-IP Kit	Thermo Fisher	88805

DynaPure XP® AllPrep DNA/RNA FFPE Kit	bphealth	BP210205S
FFPE Total Protein Extraction Kit	Sangon	C500058
**siRNA Name**		
hsa_circ_0004874 si-1	this study	TGAAGCAACTAAGAGT CCT
hsa_circ_0004874 si-3	this study	GAAGCAACTAAGAGT CCTG
**Probes**		
hsa_circ_0004874 -NB probe	this study	AGTTTCCATACACAGGA CTCTTAGTTGCT
GAPDH-NB probe	this study	TGGTGGTGCAGGAGGC ATTGCTGATGAT
