# Supplementary figures and images for: Circular SNX25 encoded radioresistance augmenter facilitates DNA damage repair in hepatocellular carcinoma by targeting BAG6-GET4 interaction
Source: Cell Death Dis. 2025 Oct 21;16(1):734. doi: 10.1038/s41419-025-08026-9 (PMC12541074; doi:10.1038/s41419-025-08026-9)

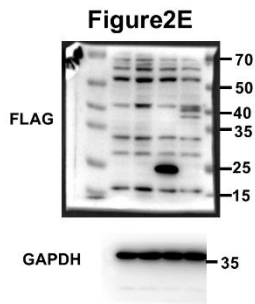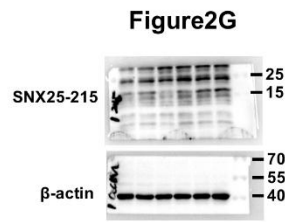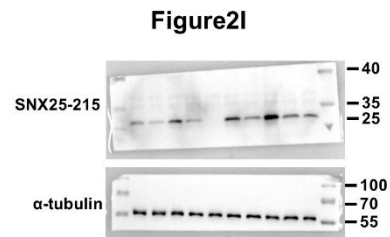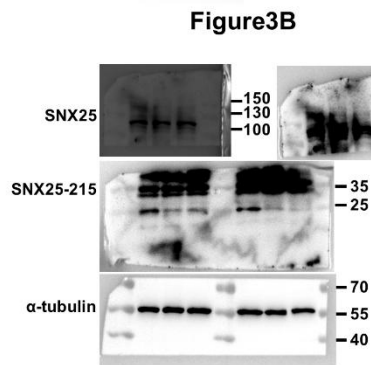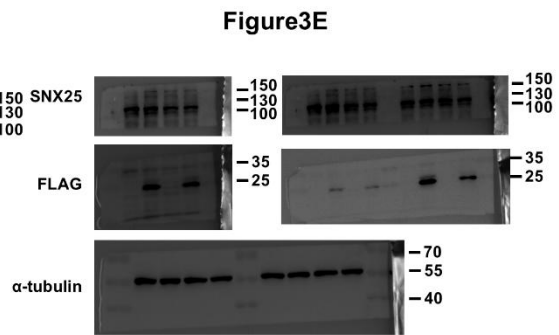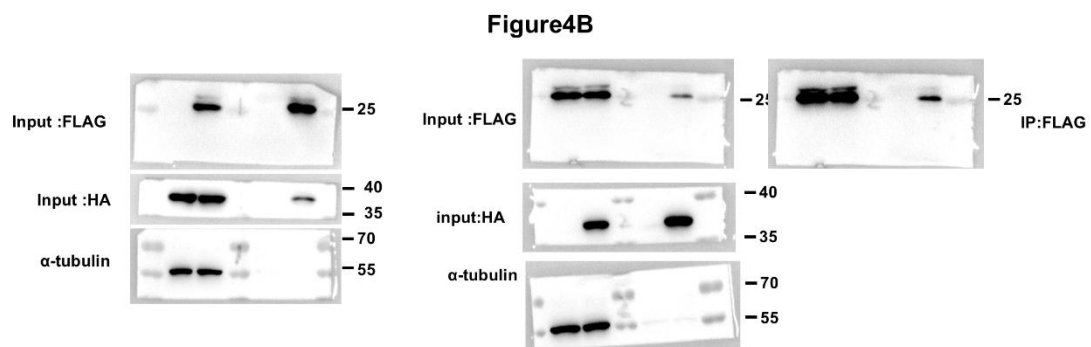

**Figure4D**

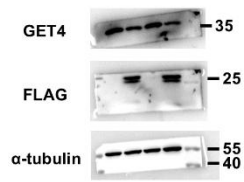

**Figure4E**

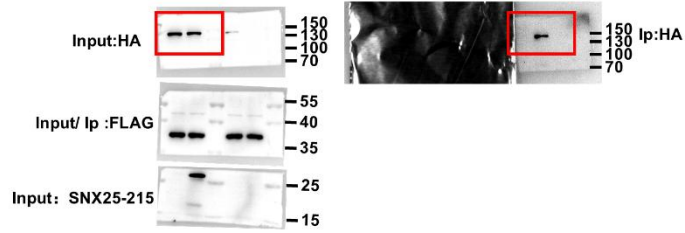

**Figure4F**

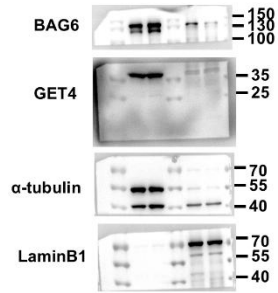

**Figure4H**

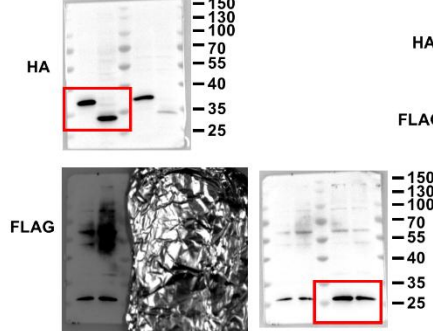

**Figure4J**

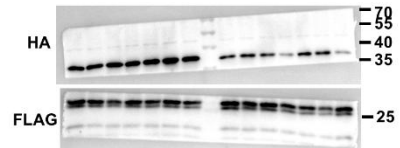

**Figure4K**

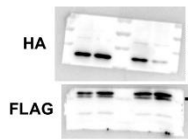

**Figure4L**

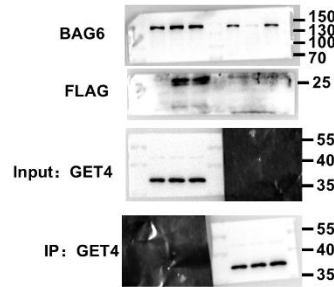

**Figure4M**

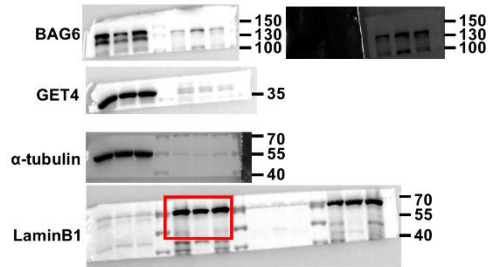

**Figure5C**

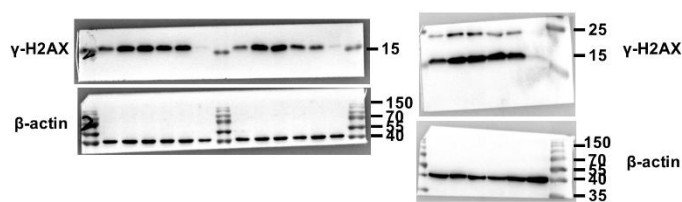

**Figure5D**

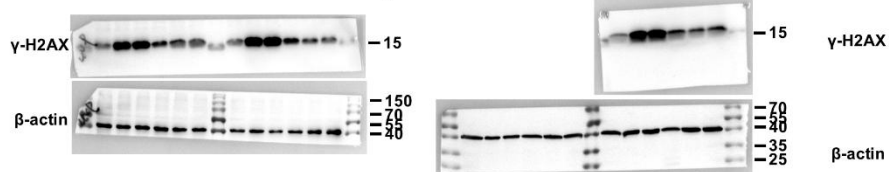

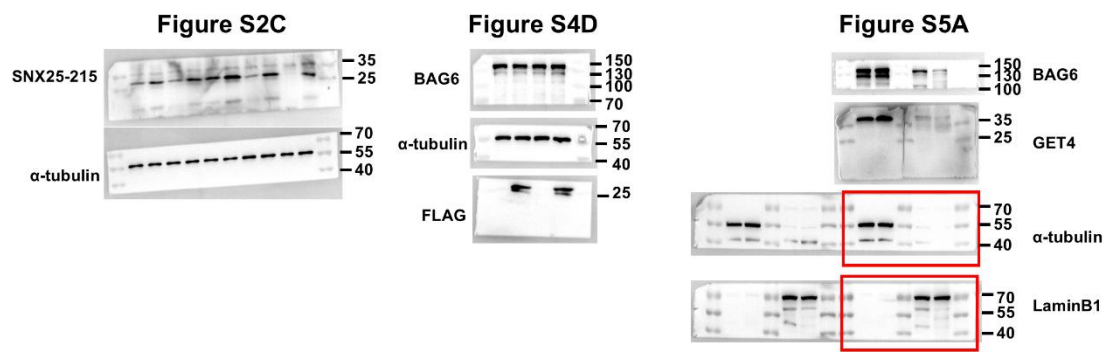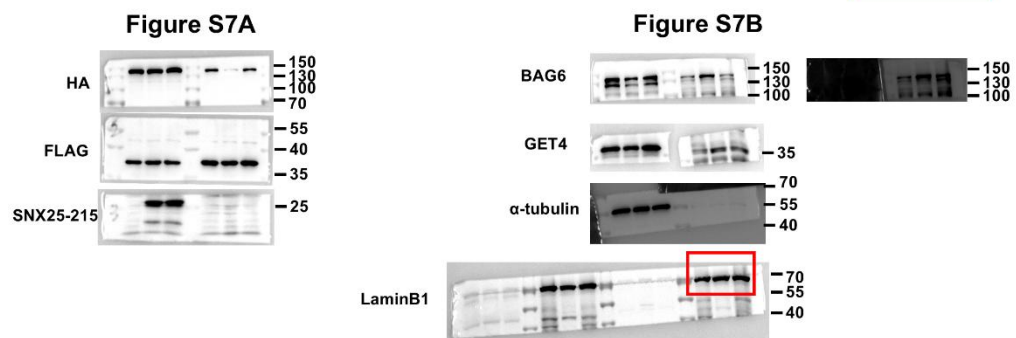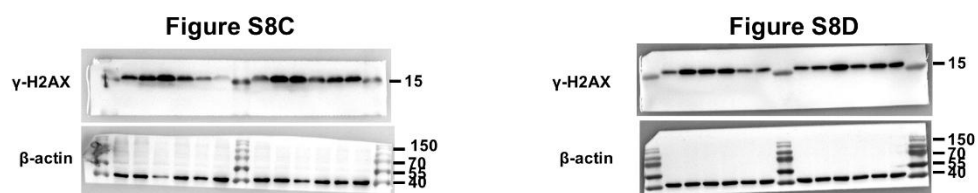

Supplement: Supplementary file 2 — Original Western blots [file 41419_2025_8026_MOESM2_ESM.pdf]
